# Food-Grade Microgels for Age-Related Macular Degeneration: Design, Fabrication, and Targeted Delivery

**DOI:** 10.3390/gels12030252

**Published:** 2026-03-17

**Authors:** Sun Ju Kim, Dong Yoon Kim, Daehyeok Jeong, Changmin Lee, Hyun-Dong Cho, Minsoo P. Kim

**Affiliations:** 1 Department of Chemical Engineering, Sunchon National University, 255 Jungang-ro, Suncheon-si 57922, Republic of Korea; sju8482@scnu.ac.kr (S.J.K.); mayday30@scnu.ac.kr (D.J.); changmin50912@gmail.com (C.L.); 2 Department of Food and Nutrition, Sunchon National University, 255 Jungang-ro, Suncheon-si 57922, Republic of Korea; zoz0329@naver.com

**Keywords:** age-related macular degeneration, food-grade microgels, nutraceutical delivery, gastrointestinal release, gut-eye axis, co-encapsulation, spatiotemporal release, precision nutrition

## Abstract

Age-related macular degeneration (AMD) is a leading cause of irreversible vision loss worldwide and is driven by complex pathophysiological processes, including oxidative stress, chronic inflammation, complement dysregulation, and vascular endothelial growth factor (VEGF)-mediated neovascularization. Nutritional interventions—particularly supplementation with carotenoids, omega-3 fatty acids, polyphenols, and essential micronutrients—have demonstrated clinical benefits in slowing disease progression, as evidenced by landmark trials such as AREDS and AREDS2. However, many AMD-relevant bioactives exhibit poor aqueous solubility, low chemical stability, and limited gastrointestinal bioavailability, which significantly constrain their therapeutic efficacy. Food-grade microgels have emerged as versatile colloidal delivery platforms capable of addressing these limitations through rational structural and physicochemical design. This review provides a systematic roadmap for developing food-grade microgels, organized into: (1) the molecular design of protein- and polysaccharide-based networks; (2) advanced fabrication strategies such as microfluidics and atomization; (3) spatiotemporal release programming within the gastrointestinal tract; and (4) multi-nutrient synergy for retinal protection. This approach highlights how controlled crosslinking, interfacial assembly, and tunable network architectures enhance nutrient stabilization. Particular emphasis is placed on spatiotemporal release programming within the gastrointestinal tract, including diffusion-limited gastric retention, pH- and bile-responsive swelling in the small intestine, and microbiota-triggered degradation in the colon. These mechanisms collectively enable region-specific release, improved micellar incorporation, enhanced systemic absorption, and more consistent retinal delivery. Furthermore, we discuss co-encapsulation strategies that accommodate both hydrophilic and lipophilic bioactives, thereby minimizing antagonistic interactions and enabling synergistic nutritional modulation of oxidative and inflammatory pathways implicated in AMD. A central novelty of this review is the integration of the gut–eye axis, framing microgel-based oral delivery as a systemic pathway to modulate retinal health via the intestinal environment. By bridging retinal disease biology with food colloid science, this review proposes food-grade microgels as a translational platform for next-generation nutraceutical interventions. The integration of programmable release behavior with clinically validated nutrient regimens offers a promising pathway toward more effective and mechanistically informed dietary management of AMD.

## 1. Introduction

Age-related macular degeneration (AMD) is the leading cause of irreversible blindness in individuals over 65 years old and remains a major contributor to global visual impairment [1]. The disease arises from a multifactorial etiology in which aging, cigarette smoking [2], alcohol consumption [3], hypertension [4], diabetes, cardiovascular dysfunction, and chronic oxidative stress [5] interact to accelerate retinal degeneration. These systemic risk factors converge on key pathological events, including progressive dysfunction of the retinal pigment epithelium (RPE), structural alterations in Bruch’s membrane, accumulation of drusen deposits [6], and dysregulation of complement activation [7]. The pathogenesis of AMD is a multifactorial process primarily initiated by chronic oxidative stress, which stems from the retina’s exceptionally high oxygen consumption and continuous exposure to photo-oxidative insults [8]. This persistent stress leads to the progressive dysfunction of the RPE and the subsequent accumulation of lipofuscin, a result of impaired phagocytosis of photoreceptor outer segments [6]. These metabolic by-products contribute to the formation of drusen—extracellular deposits situated between the RPE and Bruch’s membrane—that act as a physical barrier hindering essential nutrient transport and metabolic waste removal. Such structural and biochemical alterations foster a state of chronic para-inflammation and significant dysregulation of the complement system, specifically involving the overactivation of C3 and C5 pathways [7,9]. Eventually, chronic oxidative mitochondrial damage and local hypoxia trigger the pathological upregulation of VEGF, driving the aberrant choroidal neovascularization (CNV) and vascular leakage characteristic of advanced exudative disease [10]. Clinically, AMD encompasses a continuum from early stages to advanced forms, which the Beckman Initiative for Macular Research outlines through a five-stage classification system [11]. In its late stages, AMD manifests as either atrophic (dry) or neovascular (wet) disease, with the neovascular subtype characterized by CNV, hemorrhage, and RPE detachment—events that account for severe vision loss in nearly 80% of affected patients [9,12]. As of current 2026 estimates based on recent epidemiological data, approximately 200 million individuals worldwide are living with some form of AMD [13]. While the global burden is projected to reach nearly 288 million by 2040, this escalation is most pronounced in Asia [14,15]. By 2040, Asia is expected to account for approximately 113 million cases, representing the largest regional share of the global prevalence [14]. In the Republic of Korea, a 2024 nationwide population-based study by the SNUBH research team revealed that the prevalence of exudative AMD has increased substantially over the last decade, with projections indicating a continued sharp rise as the nation becomes a super-aging society [16]. Similarly, East Asian populations exhibit a distinct clinical profile; the Asia-Pacific PCV (polypoidal choroidal vasculopathy) Consensus Group has reported that PCV accounts for 22.3% to 54.7% of neovascular AMD cases in Asians, which is significantly higher than the 8% to 13% prevalence observed in Western cohorts [17]. Critically, this escalating global burden highlights a significant nutritional gap, as current standard-of-care pharmacotherapies primarily target late-stage neovascular complications while leaving the underlying oxidative stress in early-to-intermediate AMD largely unaddressed. This underscores the urgent need for innovative dietary and delivery strategies—such as food-grade microgels—capable of mitigating retinal oxidative stress and inflammatory damage [18,19].

**Figure 1:**
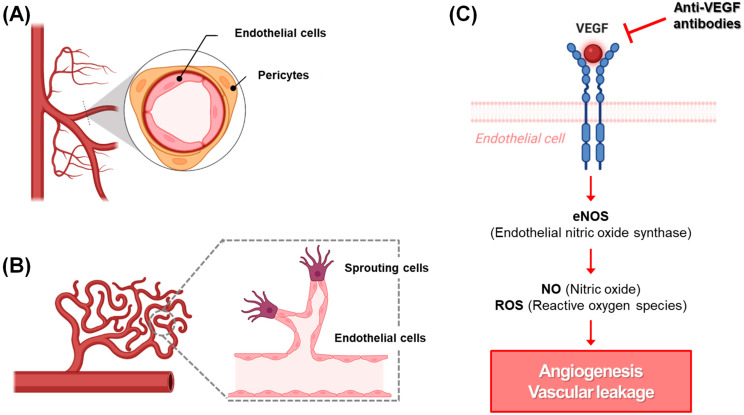
VEGF-driven angiogenesis and vascular leakage in AMD. (**A**) Structure of normal retinal microvessels. Retinal capillaries consist of endothelial cells surrounded by pericytes, forming a stable and selectively permeable vascular unit that maintains blood–retinal barrier integrity. (**B**) Pathological angiogenesis in AMD. Excessive VEGF signaling activates endothelial cells, promoting proliferation, migration, and sprouting, which leads to the formation of immature and leaky neovessels. (**C**) Anti-VEGF therapy. Anti-VEGF antibodies inhibit VEGF binding to its receptors on endothelial cells, suppressing abnormal neovascularization and reducing vascular leakage. Illustrations created with BioRender.com.

A central biological framework supporting the oral delivery of these bioactives is the gut–eye axis, a complex communication network that links intestinal homeostasis to retinal health [20]. Emerging evidence suggests that gut dysbiosis and increased intestinal permeability can exacerbate retinal inflammation and CNV through the systemic translocation of pro-inflammatory cytokines and microbial metabolites [21]. As illustrated in Figure 1, the gut–eye axis functions as a systemic pathway where the modulation of the intestinal environment directly influences the blood–retinal barrier’s integrity and oxidative stress levels in the macula [22]. By leveraging food-grade microgels to protect and target the delivery of nutrients within the gastrointestinal tract, it is possible to harness this axis to mitigate chronic retinal damage. Positioning the gut as a strategic gateway for retinal therapy provides a novel paradigm for AMD management, shifting the focus from local ocular treatments to systemic, diet-based interventions.

Current clinical management of AMD primarily relies on photodynamic therapy (PDT), laser photocoagulation, and anti-VEGF–based pharmacotherapy [19]. PDT employs intravenous administration of the photosensitizer verteporfin, which preferentially accumulates in abnormal sub-foveal vasculature. Upon irradiation at a specific wavelength, verteporfin generates reactive oxygen species that selectively damage pathological vessels and suppress VEGF-mediated angiogenesis. Laser photocoagulation similarly targets CNV through thermal ablation of aberrant vessels to reduce leakage and scar formation. Despite these mechanistic advantages, both PDT and laser photocoagulation exhibit significant limitations—most notably high recurrence rates of CNV and the risk of collateral injury to adjacent retinal tissue [23,24]. Anti-VEGF therapy has become the cornerstone for neovascular AMD (nAMD), targeting VEGF-A via intravitreal injection. Clinically established agents include bevacizumab, which inhibits all VEGF-A isoforms; ranibizumab, an antibody fragment approved for CNV prevention; aflibercept, a recombinant fusion protein with high binding affinity; and brolucizumab, a single-chain fragment with efficient retinal penetration. To further enhance clarity, we have revised the description of these agents to explicitly distinguish their molecular mechanisms. For instance, the recent clinical introduction of faricimab (Vabysmo) represents a shift toward dual-pathway inhibition, neutralizing both VEGF-A and Angiopoietin-2 (Ang-2) to improve vascular stability and extend dosing intervals [25]. Additionally, aflibercept 8 mg (Eylea HD) has been implemented to provide more sustained VEGF suppression in refractory cases [26]. Furthermore, pharmacological management extends to geographic atrophy through newly approved complement inhibitors. Pegcetacoplan (Syfovre) [27] and avacincaptad pegol (Izervay) [28] target C3 and C5 pathways, respectively, to slow the progression of retinal cell loss. Although these pharmaceutical agents substantially reduce clinical complications, they primarily target downstream angiogenic or degradative signaling and do not directly correct upstream drivers such as oxidative mitochondrial damage or chronic inflammatory stress within the RPE. This mechanistic gap underscores the need for complementary strategies capable of modulating the broader oxidative-inflammatory microenvironment.

Anti-VEGF therapy has become the cornerstone treatment for neovascular AMD (nAMD), targeting VEGF-A—the principal mediator of pathological angiogenesis—via intravitreal injection [29]. Clinically used agents include bevacizumab, ranibizumab, aflibercept, and brolucizumab [30]. Bevacizumab, a humanized monoclonal antibody originally developed for oncology, inhibits all VEGF-A isoforms by blocking their interaction with VEGFR-1 and VEGFR-2, and is widely employed off-label in ophthalmology due to its cost-effectiveness [31]. Ranibizumab, an antibody fragment approved in 2006, similarly neutralizes active VEGF-A isoforms, preventing CNV [32]. Aflibercept, approved in 2011, is a recombinant fusion protein comprising VEGFR-1/2 extracellular domains linked to an IgG1 Fc domain, offering higher VEGF-binding affinity and the added capability of neutralizing VEGF-B and placental growth factor (PlGF) [33]. More recently, brolucizumab—a 26 kDa single-chain antibody fragment lacking an Fc domain—has been shown to penetrate retinal tissues more efficiently and achieve higher intraretinal drug concentrations [34].

Although anti-VEGF agents substantially reduce the risk of severe vision loss, they present notable limitations. These therapies do not address upstream processes such as chronic inflammation, oxidative stress, or intracellular VEGF production, leaving critical elements of AMD pathophysiology untreated. Furthermore, over half of patients exhibit suboptimal or incomplete responses. Quantitatively, clinical evidence indicates that approximately 10% to 15% of patients are non-responders who show minimal anatomical improvement despite intensive anti-VEGF therapy [35]. Furthermore, the need for frequent intravitreal injections—typically every 4 to 8 weeks—coupled with high annual costs that often exceed $10,000 per patient, imposes a substantial clinical and economic burden [36]. Poor adherence to this demanding regimen frequently contributes to recurrent CNV and diminished therapeutic outcomes [37].

Figure 1 schematically summarizes the structural organization of the retinal microvasculature and the VEGF-driven pathological cascade underlying neovascular AMD. Under physiological conditions, retinal capillaries are composed of a continuous endothelial monolayer ensheathed by pericytes and supported by a specialized basement membrane, collectively forming the inner blood–retinal barrier (BRB), which tightly regulates vascular permeability and maintains retinal immune privilege [6,38] (Figure 1A). Pericyte–endothelial cell crosstalk via PDGF-B/PDGFR-β and angiopoietin–Tie2 signaling is essential for vascular stabilization and barrier integrity [39]. In nAMD, pathological upregulation of VEGF-A, largely driven by oxidative stress, hypoxia-inducible factor-1α (HIF-1α) activation, complement dysregulation, and chronic para-inflammation, results in persistent activation of VEGFR-2 on endothelial cells [7,8,10] (Figure 1B). This signaling cascade promotes endothelial proliferation, migration, cytoskeletal reorganization, and tip-cell formation, ultimately leading to aberrant CNV [12,40]. The newly formed vessels are structurally immature, poorly covered by pericytes, and exhibit disrupted tight junctions, rendering them hyperpermeable and prone to exudation. The consequent vascular leakage, subretinal fluid accumulation, and inflammatory amplification drive progressive photoreceptor degeneration and visual decline.

Although intravitreal anti-VEGF therapies effectively neutralize extracellular VEGF-A and suppress active neovascularization [29,30,31,32,33,34,37] (Figure 1C), they primarily target downstream angiogenic signaling. These agents do not directly correct upstream drivers such as oxidative mitochondrial damage, complement overactivation, metabolic dysregulation, or chronic inflammatory stress within the RPE, which are increasingly recognized as central determinants of AMD progression [7,8]. This mechanistic gap underscores the need for complementary strategies capable of modulating the broader oxidative–inflammatory microenvironment.

## 2. Nutrients for Alleviation of AMD

Nutritional intervention has gained growing scientific attention as a complementary strategy for delaying the onset and progression of AMD, primarily because it addresses upstream biological mechanisms that precede irreversible photoreceptor loss. Oxidative stress, chronic para-inflammation, impaired mitochondrial turnover, and RPE dysfunction are now recognized as central nodes in AMD pathogenesis [41]. Antioxidant vitamins—including vitamins C, E, and A derivatives—support retinal protection by neutralizing reactive oxygen species and maintaining photoreceptor viability, effects that were firmly established in the AREDS trial [42]. However, subsequent analyses have shown that the efficacy of antioxidant therapy depends strongly on nutrient bioavailability and baseline dietary intake, with AREDS2 demonstrating that carotenoid-based formulations (lutein/zeaxanthin) outperform β-carotene in safety and biological relevance [43].

Carotenoids such as lutein, zeaxanthin, and meso-zeaxanthin accumulate selectively in the macula through interactions with specific transport proteins, including scavenger receptor class B type 1 (SCARB1) and the lipid-transfer protein StARD3. Their functions extend beyond blue-light filtering to include quenching of singlet oxygen, stabilization of membrane lipids, and attenuation of photooxidative damage—mechanisms central to maintaining macular pigment optical density (MPOD), a strong biomarker of AMD risk. Similarly, long-chain omega-3 polyunsaturated fatty acids (DHA/EPA) contribute to retinal health through anti-inflammatory lipid mediator synthesis (resolvins, protectins), enhancement of membrane fluidity, and support for photoreceptor outer segment renewal [44]. Additional bioactives such as resveratrol, anthocyanins, curcumin, and zinc exhibit RPE-protective effects by modulating NF-κB signaling, complement activation, and mitochondrial redox homeostasis [45].

Despite their mechanistic importance, many of these nutrients suffer from low water solubility, extensive first-pass metabolism, and poor gastrointestinal (GI) stability. Lipophilic nutrients like carotenoids and omega-3 fatty acids rely on bile-mediated emulsification and mixed micelle formation for absorption, processes that are highly inefficient when nutrients are delivered in crystalline or aggregated forms [46]. Polyphenols undergo rapid oxidation or conjugation in the GI tract, limiting their systemic availability. These barriers highlight the need for delivery systems that improve solubilization, reduce oxidative degradation, and enhance trans-epithelial transport [47].

Food-grade microgels offer a compelling solution to these challenges by integrating structural protection with physiologically compatible release mechanisms. The hydrated polymer networks of alginate-, pectin-, or protein-based microgels physically isolate labile nutrients from pro-oxidant environments, suppress isomerization of carotenoids, and stabilize omega-3 fatty acids against lipid peroxidation. Microgels can also modulate gastrointestinal transit behavior: their tunable mesh size and interfacial architecture promote improved lipid-phase dispersion, enhance bile salt accessibility, and facilitate uniform mixed micelle formation—key determinants of intestinal absorption [48]. Importantly, the controlled swelling and enzymatic responsiveness of microgels allow nutrient release to align with peak absorption in the duodenum and jejunum, providing a level of spatiotemporal control not achievable through conventional supplementation. The coupling of established nutrient-based interventions with microgel-enabled delivery systems may substantially enhance their biological efficacy, offering a next-generation approach for nutritional modulation of AMD progression. The key nutrients implicated in AMD prevention, their biological roles, and major delivery limitations are summarized in Table 1.

The AREDS2 study has undeniably defined the current clinical paradigm for AMD nutritional intervention. However, since 2024, a new wave of clinical data and formulation technologies has begun to transcend these established boundaries. To distinguish these emerging innovations from historical standards, we have summarized the most significant advancements from 2024 to 2026 in Table 2. These recent clinical insights [49,50] emphasize that the efficacy of xanthophylls is no longer limited to age-related degeneration but extends to modern visual stressors such as digital blue light exposure. Crucially, the persistent challenge of low bioavailability—a major bottleneck for both carotenoids and polyphenols—is being systematically addressed through nanotechnology [51]. Innovations such as Chitosan Nanoparticles and Self-Nanoemulsifying Drug Delivery Systems (SNEDDS) have demonstrated the ability to increase the systemic and local bioavailability of these compounds by over 200% [52,53]. By integrating these high-efficiency delivery mechanisms, the next generation of ocular nutraceuticals can ensure that therapeutic concentrations are maintained within the retinal tissues, bridging the gap between dietary intake and clinical neuroprotection.

### Precision Nutrition: MPOD as a Clinical Compass for Personalized Delivery

The clinical landscape of 2026 has shifted toward a precision nutrition model, prioritizing interventions tailored to individual physiological biomarkers rather than standardized supplementation. Central to this approach is the assessment of MPOD, a non-invasive indicator that quantifies the concentration of lutein and zeaxanthin within the macula [41]. MPOD serves as a vital prognostic tool, as higher pigment levels are fundamentally linked to the neutralization of reactive oxygen species and the filtering of blue light, which are critical for preserving retinal integrity. The necessity for personalized delivery arises from the significant inter-individual variability in response to oral intake. Therapeutic efficacy is often hindered by complex transport mechanisms and the metabolic challenges of delivering carotenoids to the RPE [54]. Recent clinical data from 2025 indicates that low-responders—individuals with depleted MPOD—require highly efficient delivery platforms to achieve therapeutic thresholds and effectively slow the progression of geographic atrophy [49]. Food-grade microgels offer the structural versatility required to bridge this nutritional gap by providing a protective, tunable environment for lipophilic bioactives [55]. By modulating the biopolymer network and interfacial properties, microgels can be engineered to synchronize nutrient release with optimal intestinal absorption windows. This allows for a “closed-loop” delivery strategy where measured clinical data (MPOD) directly informs the design of the microgel carrier, ensuring that neuroprotective concentrations are maintained in the retinal tissues of even the most physiologically challenged patients.

## 3. Materials and Composition of Food-Grade Microgels for Targeted Delivery in AMD

Food-grade microgels are increasingly recognized as versatile carriers capable of protecting, stabilizing, and delivering bioactive nutrients relevant to AMD [56]. Their functionality is determined primarily by the intrinsic physicochemical characteristics of the biopolymers that form their three-dimensional network [57]. In particular, proteins and polysaccharides—due to their natural abundance, food safety, and tunable molecular interactions—serve as the primary structural units in microgel fabrication [58]. Their molecular architecture dictates the microgel’s gelation behavior, swelling properties, structural rigidity, loading capacity, and release mechanisms, all of which are crucial for ensuring targeted and sustained delivery of lipophilic antioxidants, carotenoids, polyphenols, and other AMD-related therapeutic nutrients [59,60,61,62]. This section provides an integrated overview of the properties of protein-based, polysaccharide-based, and hybrid microgels, and discusses how these materials support advanced delivery functions required for ocular health [63].

**Figure 2:**
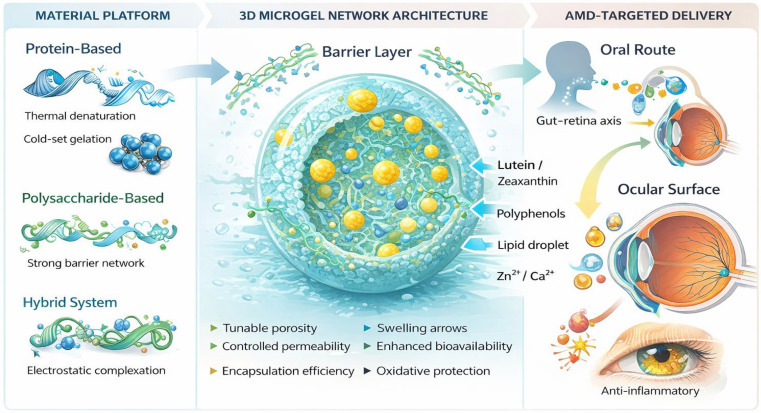
Integrated structural design of food-grade microgels for AMD-targeted nutrient delivery. Protein-, polysaccharide-, and hybrid-based microgels form hydrated 3D networks with tunable porosity and barrier properties. These architectures encapsulate carotenoids, polyphenols, lipid droplets, and mineral cofactors, providing oxidative protection and controlled release. By modulating swelling and permeability, microgels enhance bioavailability and enable AMD-focused delivery via oral (gut–retina axis) or ocular routes.

Figure 2 illustrates the integrated structural framework of food-grade microgels engineered for AMD-targeted nutraceutical delivery. These systems are constructed from protein-based matrices (e.g., heat-induced or cold-set gelled globular proteins), polysaccharide-based networks (e.g., ionotropically crosslinked alginate or pectin), or hybrid protein–polysaccharide complexes formed via electrostatic interactions [55,64,65,66,67]. The resulting hydrated three-dimensional (3D) microgel architecture is characterized by tunable mesh size, adjustable crosslinking density, and a semi-permeable barrier layer that governs mass transport kinetics. Within this network, lipophilic carotenoids such as lutein and zeaxanthin, polyphenolic antioxidants, lipid droplets, and mineral cofactors (e.g., Zn^2+^, Ca^2+^) can be efficiently encapsulated through a combination of hydrophobic interactions, hydrogen bonding, and interfacial stabilization mechanisms [55,68,69,70,71]. The polymeric matrix provides protection against oxidative degradation, enzymatic destabilization, and premature release during gastrointestinal transit.

By modulating network porosity, swelling behavior, and interfacial permeability, these microgels enable diffusion-controlled release and improved bio-accessibility of encapsulated bio-actives [55,66]. Following oral administration, enhanced micellar incorporation and intestinal uptake may support systemic transport via the gut–retina axis, whereas localized ocular formulations may facilitate direct surface delivery. Collectively, the structure–function relationships of these microgel systems underpin their potential to improve the bioavailability and therapeutic efficacy of AMD-relevant nutrients.

### 3.1. Protein-Based Microgel Matrices

Proteins possess a rich diversity of conformations—globular, fibrous, and random-coil structures—and respond sensitively to environmental conditions including temperature, pH, and ionic strength [72,73]. These features allow them to form microgels through pathways such as thermal denaturation, cold-set gelation, self-assembly, and ionic complexation [74,75,76]. Globular proteins such as whey protein isolate, β-lactoglobulin, and ovalbumin unfold when heated above their denaturation temperature, exposing hydrophobic residues and reactive thiol groups that subsequently drive aggregation into gelled micro-particles [77,78]. The resulting microgels often exhibit narrow size distributions, high water content, and soft elastic properties that can be tuned by adjusting processing variables such as heating temperature, pH relative to the isoelectric point, and the ionic strength of the solution [64,79].

These thermally induced protein microgels offer multiple advantages for encapsulating AMD-related hydrophobic compounds such as lutein, zeaxanthin, and astaxanthin [65,66,67]. Because they contain hydrophobic pockets and amphiphilic interfaces, proteins bind carotenoids and polyphenols through hydrophobic interactions, hydrogen bonding, and π-π stacking, thereby stabilizing them against photo-oxidation and enzymatic degradation during storage and digestion [68]. Cold-set gelation further expands their applicability: pre-heated proteins can be ionically crosslinked with calcium to form softer, more deformable microgels under mild conditions, which is especially advantageous for heat-sensitive antioxidants and for maintaining the integrity of nutrients during encapsulation [69].

Protein-based microgels also hold promise for ocular delivery due to their intrinsic transparency, biodegradability, and ease of surface functionalization. Their amino and carboxyl groups can be modified with targeting peptides or mucoadhesive moieties to enhance retention on ocular tissues or to enable selective binding to retinal cells [34,70]. In addition, their ability to modulate swelling in response to pH or ionic composition supports sustained release functions that are desirable for long-acting nutraceutical therapy in AMD [66,71].

**Figure 3:**
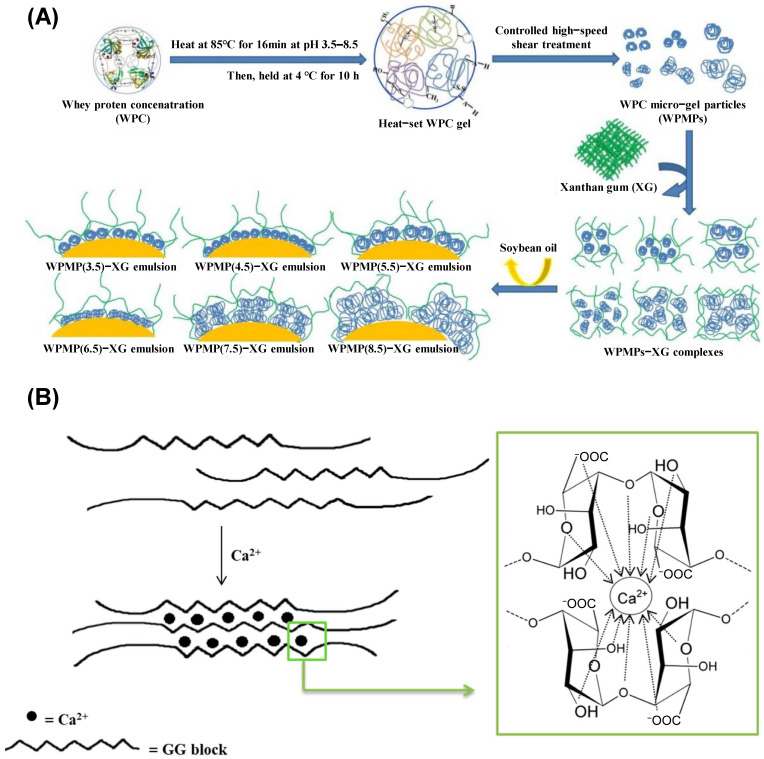
Schematic representation of protein–polysaccharide microgel formation and alginate “egg-box” crosslinking mechanism. (**A**) Whey protein concentrate (WPC) undergoes heat-induced denaturation and controlled high-shear treatment to generate whey protein microgel particles (WPMs). Subsequent electrostatic interaction with xanthan gum (XG) leads to the formation of WPM–XG complexes, which stabilize oil droplets and produce structured emulsion systems with tunable interfacial architectures. (Adapted from Ref. [67] Copyright © 2021, MDPI) (**B**) In polysaccharide-based microgels, alginate chains containing guluronic acid (GG) blocks coordinate with divalent Ca^2+^ ions to form “egg-box” junction zones. This ionic crosslinking mechanism generates a stable three-dimensional hydrogel network with enhanced mechanical integrity and barrier properties, contributing to improved protection of encapsulated bioactives. (Adapted from Ref. [80] Copyright © 2022, MDPI).

Figure 3 illustrates two representative mechanisms underlying the formation of protein–polysaccharide microgels and ionically crosslinked polysaccharide hydrogels. In protein-based systems (Figure 3A), whey protein concentrate (WPC) undergoes heat-induced denaturation, during which globular proteins partially unfold and expose hydrophobic domains and reactive sulfhydryl groups. Subsequent high-shear processing facilitates controlled aggregation, yielding whey protein microgel particles (WPMs) with defined size distribution and internal network structure. Electrostatic complexation between negatively charged xanthan gum (XG) and surface-charged WPMs leads to the formation of WPM–XG complexes, enhancing colloidal stability and interfacial viscoelasticity. These hybrid assemblies can adsorb at oil–water interfaces, stabilize emulsion droplets, and generate structured emulsion systems with tunable interfacial architectures and improved oxidative protection [67]. In contrast, polysaccharide-based microgels such as alginate hydrogels are formed through ionotropic gelation (Figure 3B). Specifically, guluronic acid (G-block) regions within alginate chains coordinate with divalent calcium ions (Ca^2+^), producing cooperative ionic junction zones described by the classical “egg-box” model. This crosslinking mechanism induces the formation of a three-dimensional hydrogel network with enhanced mechanical rigidity, reduced permeability, and improved barrier properties, thereby protecting encapsulated bioactive compounds from premature diffusion and degradation [80].

These protein-driven aggregation and polysaccharide ionotropic crosslinking strategies exemplify complementary routes for constructing structurally robust and functionally tunable microgel delivery systems.

### 3.2. Polysaccharide-Based Microgel Matrices

Polysaccharides represent another major class of biopolymers suitable for food-grade microgels. Their diverse structural motifs—linear or branched chains, variable degrees of esterification, and multiple ionizable groups—enable them to form networks through ionic, thermal, or physical interactions [48,58,60,62]. Polysaccharides vary widely in charge: alginate, pectin, carrageenan, and xanthan are typically anionic; chitosan carries a positive charge under mildly acidic conditions; starch and cellulose are largely neutral. As the selection of an optimal carrier is crucial for maximizing the therapeutic efficacy of AMD-relevant bioactives, a comparison of the most widely used polysaccharides is presented in Table 3. Alginate is highly valued for its ability to form stable hydrogels under mild conditions via Ca^2+^ mediated crosslinking, which is essential for preserving the bioactivity of heat-sensitive carotenoids and vitamins [81,82]. Chitosan, through its unique cationic properties, provides superior mucoadhesion and pH-responsive release, significantly enhancing nutrient retention at ocular and intestinal interfaces [60]. For applications requiring targeted delivery to the distal gut, pectin remains the preferred choice due to its specific degradation by colonic microbiota [63,83], while carrageenan offers the high mechanical integrity and thermal stability necessary for integration into diverse functional food systems [84]. These distinct physicochemical attributes allow for the rational design of microgels tailored to the specific absorption profiles and stability requirements of AMD-targeted nutrients.

One of the most widely used polysaccharide gelation mechanisms is calcium-mediated crosslinking, particularly in alginate and low-methoxy pectin. The so-called “egg-box” coordination between Ca^2+^ and carboxylate groups forms a strong yet biocompatible gel network with excellent stability under physiological conditions [82]. These microgels are highly effective at protecting labile carotenoids such as β-carotene, lutein, and astaxanthin from light-induced degradation and oxidative reactions—factors known to critically reduce the bioefficacy of AMD-related nutrients [56]. In addition, polysaccharide shells can form thick interfacial layers in emulsion-based microgels, enhancing physical barriers against oxygen, metal ions, and free radicals, thereby improving antioxidant preservation during food processing and storage [85].

Cationic chitosan offers unique advantages for ocular applications because of its ability to adhere strongly to negatively charged mucin layers on the corneal surface. This property supports prolonged retention of microgels at ocular interfaces, enabling more efficient nutrient absorption [86,87]. Moreover, chitosan can interact electrostatically with anionic polysaccharides such as alginate or pectin to create hybrid networks with customized permeability and mechanical stability [88,89]. These complexed gels remain stable in acidic environments but gradually swell under neutral conditions, allowing controlled release of bioactives in biological fluids. Overall, polysaccharide microgels provide robust structural protection, excellent biocompatibility, and tunable responsiveness, making them suitable for the safe delivery of hydrophobic lutein, polyphenols, minerals, and vitamins essential for AMD prevention.

### 3.3. Hybrid Protein–Polysaccharide Microgel Networks

Combining proteins and polysaccharides offers an expanded design space that leverages the complementary strengths of both material classes. For example, protein–polysaccharide complexes formed through electrostatic interactions (such as β-lactoglobulin–pectin or whey protein–chitosan) create thicker interfacial layers that improve emulsion stability and enhance encapsulation efficiency for lipophilic compounds [90]. These hybrid systems often display improved mechanical rigidity and reduced aggregation during processing compared with single-component gels [58].

Hybrid microgels can be engineered to possess dual responsiveness—for instance, swelling might be triggered by pH while permeability is influenced by ionic strength or enzymatic activity. This multiplicity of control mechanisms becomes particularly valuable for AMD-related delivery, where nutrients must survive gastrointestinal passage (in the case of oral supplements) or persist in tear fluid (in topical administration) [91]. Hybrid networks also mitigate issues such as phase separation, premature release, or structural collapse, making them promising vehicles for multi-nutrient formulations, including combinations of lutein, omega-3 fatty acids, zinc, or polyphenols [85].

In addition, hybrid matrices can incorporate lipid droplets, probiotics, or mineral ions to further expand their therapeutic functions. For instance, co-encapsulation of probiotics or polyphenols may modulate the gut–retina axis—a pathway increasingly recognized in AMD pathology [92,93,94]—while lipid cores help solubilize poorly water-soluble carotenoids and sustain their release [56,95].

### 3.4. Additional Functional Components Within Microgels

Food-grade microgels often integrate additional components that strengthen their delivery functions. Lipids or emulsified oil droplets embedded within biopolymer networks offer a favorable microenvironment for highly lipophilic nutrients, significantly improving their solubility and light stability. Solid lipid particles, nanostructured lipid cores, and Pickering-type interfaces can act as reservoirs that release carotenoids or vitamin E gradually while preventing oxidative degradation [96,97,98].

Minerals such as Ca^2+^, Zn^2+^, or Mg^2+^ may act not only as crosslinkers but also as functional co-factors for ocular health. Zn^2+^, for example, is involved in retinal enzyme function and may synergize with antioxidant nutrients delivered through microgels [99,100]. Finally, recent studies suggest that microgels containing probiotics can support anti-inflammatory pathways associated with retinal health. Such systems may complement the direct delivery of antioxidants by modulating gut microbiota profiles relevant to chronic inflammation and oxidative stress in AMD [101,102].

The choice of microgel materials profoundly influences their suitability for AMD prevention and therapy. Protein microgels offer superior transparency and favorable binding interactions with hydrophobic antioxidants. Polysaccharide microgels provide robust protection against environmental stressors and enable mucoadhesive ocular applications. Hybrid protein–polysaccharide networks integrate the strengths of both material classes and support multi-functional, multi-nutrient delivery strategies. Together, these materials form a versatile platform that can be optimized to enhance stability, bioavailability, targeted release, and therapeutic outcomes for AMD-related nutrients.

### 3.5. Molecular Interaction Mechanisms Between Microgels and AMD-Related Nutrients

Food-grade microgels often integrate additional components that strengthen their delivery functions. Lipids or emulsified oil droplets embedded within biopolymer networks offer a favorable microenvironment.

The functional efficiency of food-grade microgels in managing AMD is fundamentally governed by multi-level molecular interactions between the biopolymer matrix and the encapsulated bioactive compounds. These interactions extend significantly beyond simple physical entrapment, involving specific physicochemical forces that dictate loading capacity, chemical stability, and subsequent release kinetics [103,104]. For lipophilic nutrients such as lutein, zeaxanthin, and omega-3 fatty acids, the hydrophobic effect serves as the primary driving force for matrix association. During the fabrication of protein-based microgels, the thermal unfolding of globular proteins exposes internal non-polar amino acid residues, creating high-affinity hydrophobic pockets. These domains effectively shield sensitive carotenoids from the aqueous environment and reactive oxygen species, thereby suppressing oxidative and thermal degradation during storage and gastrointestinal transit [103,104]. In the case of essential minerals such as Zn^2+^, which acts as a critical cofactor for retinal enzymes, electrostatic coordination is the dominant interaction mechanism. Within anionic polysaccharide networks like alginate, Zn^2+^ or Ca^2+^ ions stabilize the “egg-box” junction zones of guluronic acid (GG) blocks [82]. This ionic crosslinking effectively “cages” the ions within a hydrated polymer microenvironment, which buffers the reactivity of the minerals and reduces potential gastric irritation during residence in the stomach [63,105]. Furthermore, the encapsulation of polyphenolic antioxidants such as curcumin and resveratrol involves a combination of hydrogen bonding and π–π stacking between the aromatic rings of the nutrients and the functional groups of the biopolymer chains. These interactions are highly sensitive to the local pH and ionic strength, allowing for the design of stimuli-responsive release profiles [106,107]. Specifically, the ionization state of the polymer network dictates whether these nutrient-matrix complexes remain stable under acidic gastric conditions or undergo triggered dissociation in the small intestine. By leveraging these diverse molecular forces, food-grade microgels can be engineered to prevent antagonistic interactions in multi-nutrient formulations, ensuring that each bioactive agent retains its functional potency along the gut-eye axis [108,109].

## 4. Fabrication Methods of Food-Grade Microgels for Targeted Delivery

The fabrication method plays a decisive role in determining the internal architecture, encapsulation efficiency, physicochemical stability, and release behavior of food-grade microgels [59,110]. For targeted nutritional intervention—particularly for the delivery of hydrophobic carotenoids, polyphenols, omega-3 fatty acids, and antioxidant micronutrients relevant to AMD—the microgel production technique must ensure (i) structural fidelity under physiological conditions, (ii) minimal degradation of bioactives, and (iii) tunable microstructure suitable for controlled release [93,94,111,112,113].

**Figure 4:**
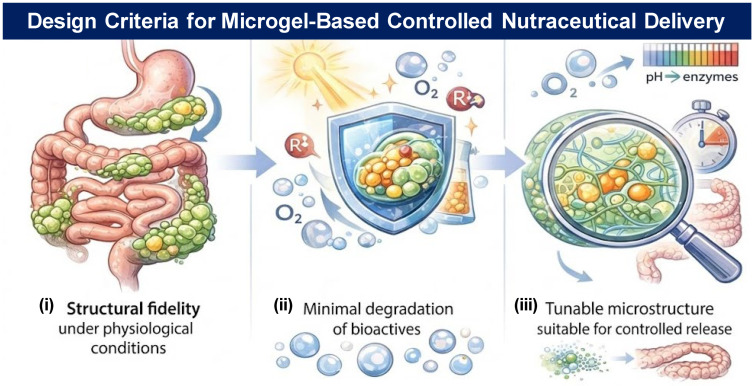
Schematic illustration of the key performance requirements for microgel fabrication in controlled nutraceutical delivery systems. (**i**) Microgels must maintain structural fidelity under physiological conditions (e.g., gastrointestinal pH, enzymes, and mechanical stress) to preserve their integrity during transit. (**ii**) The encapsulated bioactive compounds should exhibit minimal degradation, protected from oxygen, light, and reactive species to ensure high stability and functionality. (**iii**) The microgel microstructure must be tunable—such as pore size, crosslinking density, and network architecture—to enable precise, controlled release profiles in response to environmental triggers within the gastrointestinal tract.

Figure 4 summarizes the principal fabrication strategies employed for constructing food-grade microgels with controlled size distribution, internal architecture, and functional performance. Contemporary approaches can be broadly categorized into microfluidic templating, spray-based atomization processes, and high-energy homogenization techniques. Microfluidic platforms enable the generation of monodisperse droplets with precisely tunable geometries through controlled laminar flow and shear modulation (Figure 4i), allowing for uniform crosslinking and highly reproducible microgel formation [110,114,115]. These systems facilitate fine control over particle diameter, internal compartmentalization, and encapsulation efficiency, making them particularly suitable for precision nutrient delivery. Spray-drying and microfluidic-jet spray drying methods provide scalable routes for transforming emulsified or hydrogel precursors into dry microgel particles while preserving encapsulated bio-actives [116,117] (Figure 4ii). Process parameters such as inlet temperature, atomization pressure, and feed composition critically influence particle morphology, porosity, and oxidative stability. Additionally, advances in microgel engineering at the molecular and colloidal scales have expanded the functional versatility of these systems, enabling programmable responsiveness and structural tunability [118] (Figure 4iii). These fabrication platforms offer complementary advantages in terms of scalability, structural precision, and bioactive protection, thereby supporting the rational design of nutraceutical delivery systems.

Current manufacturing routes can be broadly categorized into physicochemical assembly methods, which rely on intrinsic biopolymer interactions, and mechanically driven structuring techniques, which utilize external forces or templating systems to define droplet size and morphology [73,110,114,118]. Each method confers unique advantages, and understanding these distinctions is essential for designing microgels optimized for AMD-focused nutraceutical delivery [61].

The fabrication method plays a decisive role in determining the internal architecture, encapsulation efficiency, physicochemical stability, and release behavior of food-grade microgels [55]. For targeted nutritional intervention, the production technique must ensure structural fidelity under physiological conditions, minimal degradation of bioactives, and tunable microstructure suitable for controlled release. Each method confers unique advantages, and understanding these distinctions is essential for designing microgels optimized for AMD-focused nutraceutical delivery [64]. To provide a practical roadmap for industrial selection, Table 4 compares physicochemical and mechanical fabrication strategies across key performance indicators. While mechanical methods offer superior scalability and particle size control, physicochemical routes are often preferred for highly sensitive antioxidants due to their mild processing conditions. Specifically, for heat-sensitive bioactives such as lutein, “cold-processing” mechanical alternatives—including spray chilling or electrospraying—should be prioritized over conventional spray drying to prevent thermal degradation and preserve functional potency [88,89,117,119].

### 4.1. Physicochemical Approaches

Physicochemical methods generate microgels by inducing molecular association, conformational transitions, or electrostatic interactions within or between biopolymers. These methods often operate under mild processing conditions, which is advantageous for protecting antioxidants and carotenoids vulnerable to thermal or oxidative degradation [59,118]. Although each physicochemical route differs in its underlying mechanism, all share the capacity to produce microgels with tunable porosity, hydration, and barrier properties. Self-association is one of the most versatile routes for microgel formation because it leverages the natural responsiveness of biopolymers. For globular proteins such as whey protein isolate (WPI), β-lactoglobulin, or ovalbumin, heating above their denaturation temperatures unfolds tertiary structures and exposes hydrophobic residues and thiol groups. These domains aggregate through hydrophobic interactions and disulfide bonding to form colloidal protein microgels with particle sizes ranging from tens of nanometers to several micrometers, depending on pH, protein concentration, and ionic strength [72,73]. Importantly, these hydrophobic pockets and amphiphilic surfaces provide a favorable microenvironment for entrapping highly lipophilic AMD-related compounds such as lutein and zeaxanthin, thereby preventing oxidative degradation during storage and digestion. Cold-set gelation provides a complementary alternative for heat-sensitive bioactives. In this process, proteins are first thermally denatured under controlled conditions and then rapidly cooled. Subsequent crosslinking—often induced by Ca^2+^—results in microgels with softer textures and narrower size distributions [120,121]. These cold-set microgels maintain high encapsulation efficiencies while avoiding additional thermal stress, making them particularly suitable for carotenoids and antioxidant polyphenols. Polysaccharides, including alginate, pectin, and gellan gum, undergo self-assembly primarily through ionic crosslinking. For instance, alginate forms “egg-box” junction zones with Ca^2+^, producing hydrogel networks with excellent biocompatibility and structural stability [82]. These networks exhibit strong barrier properties against oxygen and light exposure—both major causes of carotenoid degradation—highlighting their suitability for encapsulating sensitive AMD-related bioactives.

Associative complexation, frequently manifested as complex coacervation, results from electrostatic attraction between oppositely charged polymers. Examples include systems such as whey protein–pectin, gelatin–gum arabic, or casein–alginate. When charge densities and environmental conditions are appropriately tuned, phase separation produces dense polymer-rich droplets that solidify into microcapsules with a characteristic core–shell architecture [122]. Because coacervation typically occurs at room temperature or mildly acidic pH, it is exceptionally well suited for encapsulating fragile antioxidants and probiotics. The shell layer formed by complex coacervation often exhibits superior oxygen barrier properties, which is crucial for enhancing the photostability and oxidation resistance of carotenoids. Furthermore, the permeability of the coacervate membrane can be tailored by adjusting polymer ratios, ionic strength, or pH, enabling controlled release in physiological environments relevant to oral delivery or intestinal absorption [123].

**Figure 5:**
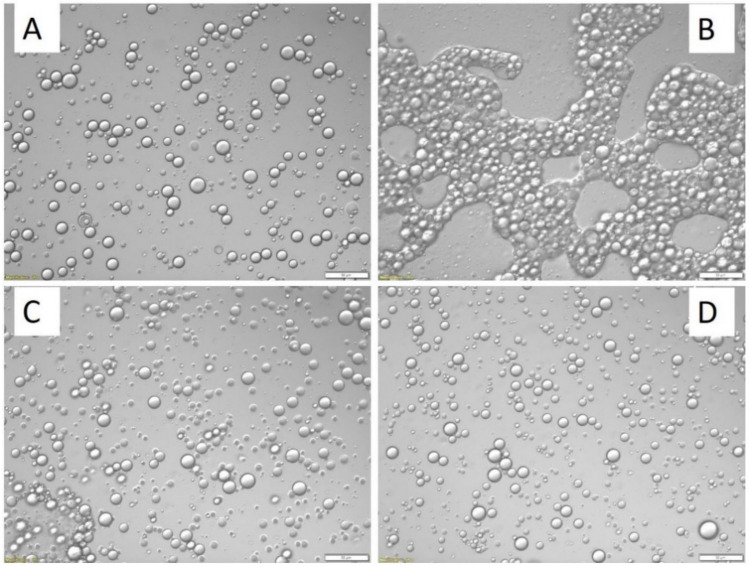
Optical microscopy images of process steps during complex coacervation of peppermint essential oil: oil-in-water emulsion at pH > 5 (**A**), coacervation at pH = 4.2 (**B**), crosslinking at pH 9 (**C**) and crosslinked capsules (**D**). The scale bar corresponds to 50 µm. (Adapted from Ref. [124] Copyright © 2021, MDPI).

Figure 5 illustrates the sequential physicochemical transitions occurring during complex coacervation-based microencapsulation of peppermint essential oil. At alkaline conditions (pH > 5), an oil-in-water (O/W) emulsion is initially formed, where dispersed oil droplets are stabilized by adsorbed biopolymers at the oil–water interface (Figure 5A). Upon gradual acidification to pH 4.2, electrostatic attraction between oppositely charged biopolymers induces phase separation, leading to the formation of a polymer-rich coacervate phase that preferentially deposits around the oil droplets (Figure 5B). This coacervate layer progressively thickens, forming a viscoelastic interfacial film that encapsulates the hydrophobic core. Subsequent adjustment to alkaline conditions (pH 9) facilitates chemical crosslinking of the coacervate matrix, thereby reinforcing the capsule shell structure and enhancing mechanical stability (Figure 5C). The final crosslinked microcapsules exhibit well-defined spherical morphology and improved barrier properties against diffusion and oxidative degradation [124] (Figure 5D). This stepwise pH-triggered coacervation and crosslinking process exemplifies a versatile encapsulation strategy for volatile and oxidation-sensitive bioactives in food-grade delivery systems.

In mixtures of incompatible biopolymers—such as gelatin/gellan, starch/pectin, or whey protein/polysaccharide blends—thermodynamic phase separation provides another effective microgel fabrication route. Under defined temperature or compositional conditions, the system segregates into polymer-rich and polymer-poor phases. The polymer-rich droplets subsequently undergo gelation through cooling or ionic crosslinking, forming uniform microgels with controlled internal morphology [64,115]. Because this process accommodates both hydrophilic and hydrophobic compounds, it supports the co-delivery of complex nutrient combinations relevant to AMD prevention, such as lutein, zinc, and omega-3 fatty acids.

### 4.2. Mechanical Approaches

Mechanical fabrication techniques manipulate biopolymer solutions using extrusion, atomization, emulsification, or microfluidic structuring to produce microgels with specific sizes and shapes. These techniques are especially valuable for industrial scalability and achieving monodisperse particle populations.

**Figure 6:**
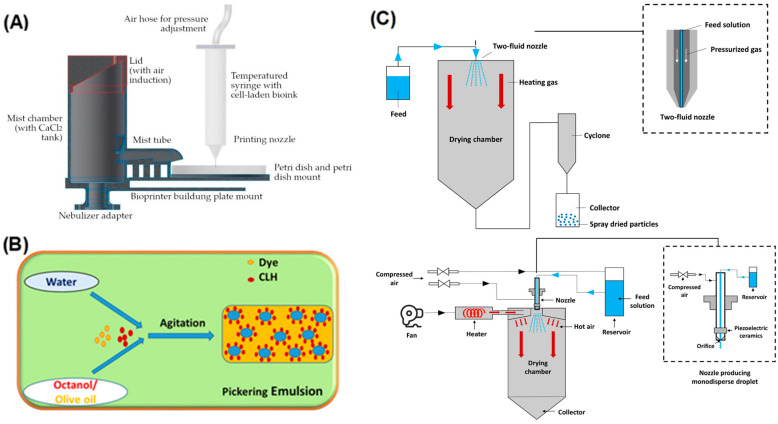
Representative mechanical fabrication strategies for food-grade microgels. These approaches utilize externally applied mechanical forces and droplet structuring techniques to regulate particle size, morphology, and internal architecture. (**A**) Extrusion-based gelation, where polymer solutions are forced through a nozzle into a crosslinking bath to form hydrogel beads. (Adapted from Ref. [13] Copyright © 2018, MDPI) (**B**) Emulsion-templated gelation, in which dispersed droplets serve as templates for microgel formation through interfacial polymer stabilization and subsequent solidification. (Adapted from Ref. [125] Copyright © 2024, MDPI) (**C**) Spray drying, a scalable atomization-based technique that converts liquid emulsions or hydrogel precursors into dry microgel particles via rapid solvent evaporation. (Adapted from Ref. [117] Copyright © 2021, MDPI).

As illustrated in Figure 6, mechanically assisted fabrication strategies constitute a major class of techniques for producing food-grade microgels with controlled particle size, morphology, and internal architecture.

In Figure 6A, extrusion-based gelation is depicted, where a biopolymer precursor solution is forced through a nozzle into a crosslinking bath—commonly containing multivalent cations such as Ca^2+^—to induce rapid ionotropic gelation and generate hydrogel beads. The final particle diameter and structural uniformity are governed by processing parameters including nozzle diameter, extrusion rate, and crosslinker concentration [125]. Extrusion involves dispensing biopolymer solutions through a nozzle into a crosslinking bath, usually containing CaCl_2_. Upon contact, droplets instantly undergo ionic gelation, forming hydrogel beads with well-defined morphology. Conventional extrusion produces beads on the millimeter scale; however, micro-extrusion and vibrating-nozzle technologies now allow the formation of microgels in the 50–200 μm range [126]. Although larger than typical ocular carriers, these microgels play a significant role in oral delivery systems targeting systemic absorption routes, including the gut–retina axis, which is increasingly implicated in AMD pathology.

Figure 6B illustrates emulsion-templated gelation, in which dispersed droplets act as confined reaction microenvironments. Biopolymers adsorb at the oil–water interface and subsequently undergo ionic, thermal, or chemical crosslinking, yielding microgel particles with tunable interfacial architecture and enhanced encapsulation efficiency for lipophilic bioactives [117]. Emulsion-templated gelation is one of the most versatile and widely studied microgel fabrication methods. In these systems, dispersed droplets in an emulsion serve as precursors for microgels, which solidify through ionic crosslinking, enzymatic reactions, pH shifts, or heating. O/W, W/O, and W/O/W emulsions allow simultaneous encapsulation of hydrophilic and hydrophobic compounds. Pickering emulsions, stabilized by solid particles such as cellulose nanocrystals, provide remarkable resistance to coalescence and oxidation, making them highly effective for carotenoid encapsulation [127,128]. Double emulsions further allow encapsulation of multiple therapeutic agents requiring distinct release kinetics—a valuable property for AMD, which involves both oxidative stress and inflammation [129,130]. High-shear homogenization of protein solutions can generate microparticulated protein assemblies with internal hydrophobic cavities [131,132]. These particles mimic fat-like textures but also function as carriers for lipophilic compounds. Although their internal structure is less uniform compared with microfluidic microgels, their scalability and compatibility with dairy- or protein-based formulations make them attractive for functional foods enriched with retinal-protective antioxidants.

Atomization—including spray drying, spray chilling, and spinning-disk atomization—enables rapid conversion of emulsions or biopolymer solutions into dry microgels [116]. Spray drying produces particles ranging from 1 to 20 μm and is widely used in the food industry due to its scalability and cost-efficiency. As shown in Figure 6C, spray drying represents a scalable atomization-based strategy that transforms liquid emulsions or hydrogel precursors into dry microgel particles via rapid solvent evaporation under controlled thermal conditions. Critical parameters such as inlet temperature, feed composition, and atomization pressure influence particle porosity, surface morphology, and oxidative stability of encapsulated compounds [121]. Spray drying is widely recognized as one of the most scalable techniques for producing dried microgel powders suitable for industrial nutraceutical formulations [81,133]. However, the process exposes encapsulated compounds to elevated temperatures that may induce thermal degradation of heat-sensitive bioactives. Typical spray-drying operations involve inlet air temperatures of approximately 140–200 °C, while outlet temperatures are generally maintained between 60 and 90 °C depending on feed composition and moisture removal requirements [134,135]. Although the residence time of droplets in the drying chamber is relatively short (typically 1–10 s), heat-sensitive nutraceuticals such as carotenoids, polyphenols, and certain vitamins may still undergo oxidative or thermal degradation during atomization and drying [81]. For example, carotenoid losses during spray drying have been reported to range from approximately 10–40%, depending on formulation composition and processing [38,136]. To mitigate these risks, the use of wall materials with high glass transition temperatures (T_g_) and the incorporation of antioxidant-rich protein-polysaccharide blends are essential to shield the core bioactives from direct thermal exposure and rapid oxidation at the air-water interface [81]. Spray chilling, in contrast, solidifies droplets through cooling rather than heat, reducing thermal stress and improving the retention of heat-sensitive antioxidants like lutein. Similarly, spinning-disk atomizers generate droplets with narrow size distributions, offering precise control over microgel morphology. Overall, atomization methods are ideal for generating storage-stable powdered microgels for nutritional formulations targeting AMD.

Microfluidic devices generate highly monodisperse droplets that can be crosslinked in situ to form microgels with precisely defined sizes (10–500 μm), morphologies, and internal architectures. The laminar flow environment ensures low shear stresses, preserving bioactive integrity. Microfluidic routes enable advanced designs such as core–shell microgels, Janus particles, and stimuli-responsive carriers tailored to pH, ionic strength, or enzymatic activity [110,114]. Although throughput remains a limitation, parallelized microfluidic arrays and droplet generators are rapidly improving scalability. The ability to co-encapsulate multiple AMD-related nutrients—such as lutein and omega-3 fatty acids—makes microfluidic microgels a promising technology for precision retinal nutrition.

Micromolding and lithography, including PRINT (Particle Replication In Non-wetting Templates) and soft lithography, allow fabrication of microgels with highly defined geometries and programmable degradation profiles. While traditionally used in biomedical engineering, applying food-safe biopolymers such as alginate, gelatin, or modified starch enables the creation of structured microgels with enhanced mucoadhesion or directional release characteristics [137,138]. Such geometric control may further improve retention or targeting efficiency in ocular and gastrointestinal pathways.

### 4.3. Comparative Evaluation and Selection Criteria for AMD-Targeted Delivery

The selection of an optimal microgel fabrication method involves a strategic balance between structural precision, nutrient stability, and industrial feasibility. Although various strategies exist, their suitability is dictated by the physicochemical sensitivities of the encapsulated bioactives (Table 4).

- Ionic Gelation: This is widely regarded as one of the most appropriate strategies for AMD-related carotenoids like lutein and zeaxanthin. Its primary advantage lies in mild processing conditions (ambient temperature and aqueous media), which effectively prevent the thermal isomerization and oxidative degradation of sensitive xanthophylls [81,103]
- Spray Drying: Despite the risk of thermal stress, this remains the most commercially viable method for large-scale production. It is particularly suitable for generating storage-stable, fortified powders for multi-nutrient formulations, provided that appropriate wall materials are used to shield the core [134].
- Complex Coacervation: This method provides exceptional oxidative protection through a dense biopolymer shell, making it ideal for highly sensitive polyphenols. However, its sensitivity to pH and ionic strength can limit its application in complex food matrices [122].
- Emulsion Templating and Microfluidics: These strategies offer the highest level of structural control and monodispersity, which is essential for precision nutrition and studying the gut-eye axis. However, low throughput remains a primary bottleneck for mass-market interventions [97,110,114].

For food-grade AMD nutraceuticals, ionic gelation and spray drying are highlighted as the most practical and effective methods due to their balance of high encapsulation efficiency, process simplicity, and scalability.

## 5. Encapsulation Strategies

Encapsulation is the central design function of food-grade microgels [72,139]. In the context of precision nutrition and functional foods, microgels must (i) physically isolate sensitive bioactive agents from harsh gastric and early intestinal conditions, (ii) modulate spatiotemporal release so that payloads are delivered to defined regions of the gastrointestinal (GI) tract, and (iii) improve uptake and bioavailability without compromising food safety or sensory quality [61,102]. Microgels, broadly defined as crosslinked, water-swollen polymer networks in the roughly 10 nm to 100 µm size regime, can be chemically or biologically modified to improve biocompatibility, tune mechanical resilience, and control diffusion and degradation behavior [118].

**Figure 7:**
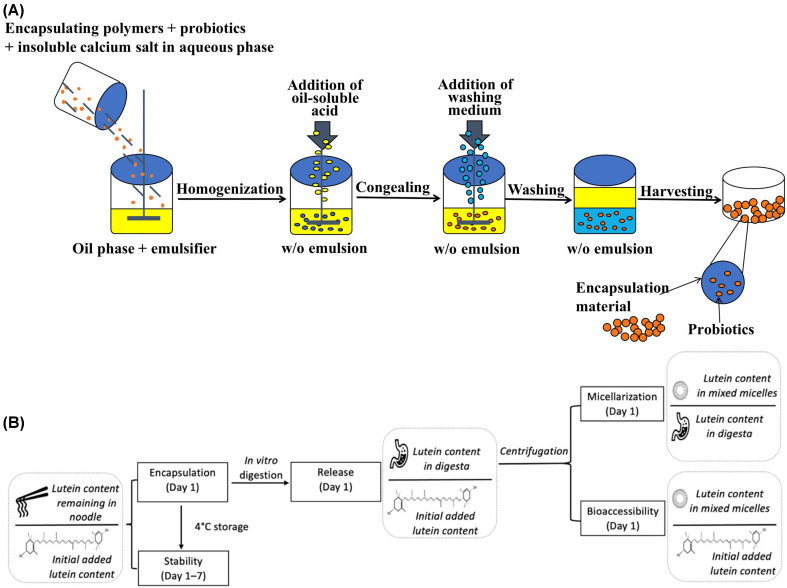
Schematic representation of microgel encapsulation strategies for different classes of payloads and their gastrointestinal transit behavior. (**A**) Multilayer microgel system designed to protect probiotic cells from gastric acid exposure and enable pH-triggered release in the intestine (adapted from Ref. [140] Copyright © 2019, Frontiers Media SA). (**B**) Hydrogel-entrapped lipophilic droplets exhibiting enhanced physicochemical stability and improved mixed-micelle formation during small intestinal digestion. (Adapted from Ref. [141] Copyright © 2021, MDPI).

Figure 7 schematically depicts representative microgel encapsulation strategies tailored for distinct classes of bioactive payloads and their gastrointestinal (GI) transit behavior. As illustrated in Figure 7A, multilayered microgel architectures are designed to protect probiotic microorganisms from the highly acidic gastric environment. In these systems, probiotic cells are encapsulated within ionically crosslinked alginate cores and further reinforced by chitosan or polyelectrolyte coatings, forming a diffusion-limiting barrier against gastric acid penetration. Upon transition to the higher pH conditions of the small intestine, partial deprotonation and matrix swelling facilitate pH-triggered release, thereby improving cell viability and colonization potential [140]. In contrast, Figure 7B demonstrates hydrogel-based encapsulation of lipophilic micronutrients or oil droplets within biopolymeric matrices. During simulated gastrointestinal digestion, the hydrogel network stabilizes dispersed lipid phases and modulates lipase accessibility, thereby enhancing lipid digestion kinetics and promoting the formation of mixed micelles necessary for intestinal absorption. This strategy improves physicochemical stability and bioaccessibility of carotenoids and other hydrophobic nutraceuticals [141].

In food systems, these microgels are commonly constructed from biopolymers such as alginate, pectin, gelatin, whey proteins, or mixed polysaccharide–protein networks. They are fabricated by emulsification, spray-drying, ionotropic gelation, or increasingly by microfluidic droplet generation, which enables highly monodisperse, core–shell architectures. The hydrogel-like internal network provides high water content, mechanical compliance, and a controllable mesh size while still functioning as a selective diffusion barrier, allowing the encapsulated cargo to survive gastric transit and instead be released under pH, enzymatic, or microbial triggers in the small intestine or colon.

### 5.1. Encapsulation of Probiotics

Probiotic microorganisms are among the most demanding cargos for any ingestible delivery system: they must remain viable during processing and storage, resist acid and bile exposure in the stomach and duodenum, and then colonize the distal gut to exert metabolic, immunomodulatory, or barrier-repair functions [61,102]. Food-grade microgel systems for probiotics respond to these constraints using polysaccharide- and protein-based matrices that act as physical shelters (“silkworm cocoons”), typically surrounding the bacterial cells with one or more protective layers, or as networked gel environments (“spider webs”), in which the bacteria are embedded in a hydrated 3D mesh that limits diffusive access of acid, oxidants, and bile salts.

A common strategy is the formation of ionically crosslinked alginate microgels, often further coated with secondary biopolymers such as chitosan or pectin to build multilayer shells with pH-responsive behavior [142]. Recent in vitro studies (2024–2025) have quantified the efficacy of these architectures, demonstrating that multilayered beads can maintain probiotic viability above 85% after 2 h of exposure to simulated gastric fluid (pH 2.0), whereas non-encapsulated cells show near-total depletion [142,143]. These multilayered coatings reduce proton and bile penetration in simulated gastric fluid, thereby preserving cell viability, and subsequently swell or partially disassemble at near-neutral intestinal pH to permit controlled release and colonization in the lower gut. Inclusion of buffering microdomains (for example, carbonate phases) or lipid droplets within the same microgel can further suppress local acidification and slow the ingress of harmful species, which improves survival through the stomach while deferring release until later intestinal segments where colonization is beneficial. In addition to pH protection, surface charge engineering of the outer shell can be exploited to enhance mucosal adhesion. For example, cationic or thiolated polysaccharide coatings can interact electrostatically or via disulfide exchange with negatively charged colonic mucins, which prolongs residence time in inflamed or dysbiotic regions and supports more localized therapeutic action [143]. Taken together, these encapsulation strategies translate pharmaceutical principles of protection, site-specific delivery, and mucosal localization into fully food-grade probiotic formats, potentially modulating the gut–retina axis for AMD management.

### 5.2. Encapsulation of Lipophilic Vitamins and Other Hydrophobic Micronutrients

Fat-soluble vitamins (A, D, E, K), carotenoids, polyphenols, and other lipophilic nutraceuticals present two fundamental challenges: they are chemically labile (susceptible to oxidation, isomerization, and thermal degradation), and they display intrinsically poor aqueous solubility, which limits absorption in the small intestine. Microgel encapsulation directly addresses both issues [142,144,145]. Current experimental data for AMD-relevant lipophiles like lutein and zeaxanthin indicate that microgel-templated systems preserve over 80% of the initial bioactive content after accelerated oxidative stress tests and simulated digestion [60,141]. When these microgel capsules erode or swell in the small intestine, bile salts and lipases can more efficiently emulsify and solubilize the encapsulated hydrophobes into mixed micelles, leading to bioaccessibility rates exceeding 75%, a significant improvement over the <20% observed in native forms. In one widely used approach, hydrophobic actives are incorporated into submicrometer or micron-scale oil droplets and then immobilized within a protein–polysaccharide gel network—for example, whey protein or gelatin complexes crosslinked within an alginate or pectin matrix—forming a core–shell or filled-gel morphology [97].

The interfacial layer surrounding each droplet and the surrounding gel matrix act as coupled diffusion and steric barriers that limit oxygen and pro-oxidant access, thereby suppressing oxidative and thermal degradation during processing and storage [130,142]. At the same time, dispersing lipophilic bioactives within monodisperse droplets trapped in a hydrogel dramatically increases their effective interfacial area during digestion. When these microgel capsules erode or swell in the small intestine, bile salts and lipases can more efficiently emulsify and solubilize the encapsulated hydrophobes into mixed micelles, which improves bioaccessibility and ultimately increases systemic bioavailability.

By tuning shell thickness, internal oil fraction, and the degree of ionic or thermal crosslinking, release of these lipophilic actives can be delayed until the duodenum or jejunum—where lipid uptake is physiologically optimized—rather than occurring prematurely in the stomach where acid-catalyzed degradation and gastric emptying dynamics would otherwise reduce efficacy [105,146]. This coupling of interfacial stabilization and programmed GI release is particularly attractive for precision nutrition, where lipid-soluble micronutrients must be delivered at effective local doses while minimizing off-target irritation or loss of activity.

### 5.3. Encapsulation of Minerals and Metabolic Cofactors

Essential minerals (e.g., Ca^2+^, Fe, Zn^2+^) and low-molecular-weight cofactors can be irritants in free form, especially in the stomach, and can also exhibit low uptake efficiency when delivered as unprotected salts [100]. Food-grade microgels provide an effective route to coordinate or chelate these ions within a hydrated polymer network, such as an alginate-block matrix crosslinked by divalent cations or a protein-based network with defined binding sites [105]. In this design, the mineral is effectively “caged” in a soft, water-rich microenvironment, which buffers direct exposure to the gastric mucosa and reduces acute irritation during gastric residence [147].

Specifically, in alginate-based systems, Ca^2+^ or Zn^2+^ ions stabilize the “egg-box” junction zones of guluronic acid (GG) blocks, significantly enhancing the mechanical integrity and barrier properties of the particle [82]. As the microgel swells, erodes, or is enzymatically loosened under the near-neutral pH and enzyme-rich conditions of the proximal small intestine, the coordinated ions are gradually released in a more bioavailable and more physiologically tolerable form [61]. Functionally, this is analogous to controlled-release pharmaceutical depots, but implemented entirely with Generally Recognized As Safe (GRAS) food biopolymers and food-compatible gelation chemistries [70]. This regulatory alignment ensures that mineral-fortified microgels can be rapidly integrated into dietary regimens without the protracted approval cycles typical of synthetic carriers.

### 5.4. Structure–Function Design Principles

Although the specific payloads differ—live probiotics, hydrophobic vitamins and polyphenols, or ionic cofactors—the structural logic of food-grade microgel encapsulation is consistent. Microgels behave as soft, crosslinked reservoirs whose mesh size, surface chemistry, interfacial architecture, and overall size distribution can be engineered to control (i) loading efficiency, (ii) retention under gastric and duodenal stress, and (iii) spatially programmed release at target GI sites [61]. Microfluidic production is especially powerful because it can generate highly monodisperse droplets or core–shell capsules in the 10–100 μm range with tightly controlled shell thickness, internal oil fraction, and biopolymer composition [114]. These parameters directly dictate how rapidly acid, bile salts, and reactive oxygen species penetrate the capsule; how strongly the particle resists deformation, erosion, or fracture under peristaltic shear; and how precisely the capsule ruptures, swells, or releases cargo in response to local pH, enzymatic digestion, or bile-driven lipid solubilization [100]. In parallel, surface functionalization—for example, imparting positive charge or thiol-containing moieties to promote mucoadhesion—can be leveraged to extend residence time at specific mucosal sites (e.g., inflamed colon) while maintaining food-grade status [86]. Viewed from a translational perspective, these encapsulation strategies position food-grade microgels as programmable, ingestible depots. They physically isolate sensitive actives, escort them through gastric and proximal intestinal stress, and then deploy them with spatial and temporal precision at sites where they can modulate appetite signaling, restore micronutrient balance, or act on disease-relevant tissues [70]. This convergence of materials engineering, digestive physiology, and nutritional function is a defining feature of next-generation “precise nutrition” platforms, highlighting the potential for translating microgel systems toward clinically meaningful dietary interventions for AMD management [94].

## 6. Release Mechanisms

**Figure 8:**
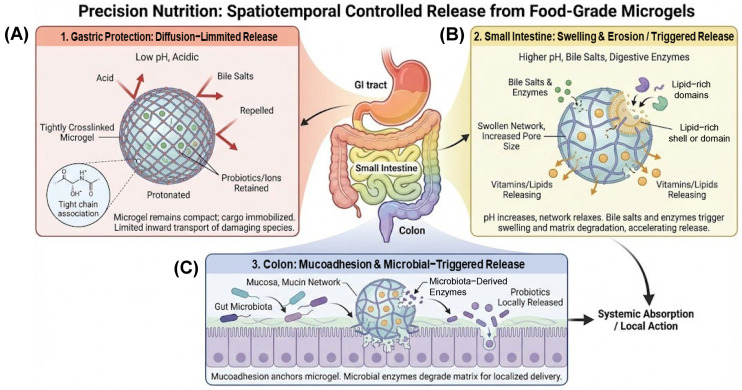
Spatiotemporal controlled release behavior of food-grade microgels along the gastrointestinal (GI) tract. (**A**) Gastric protection via diffusion-limited retention within tightly crosslinked networks. (**B**) Small intestinal release driven by pH-induced swelling, bile salt interaction, and matrix erosion, facilitating mixed micelle formation and enhanced lipophilic nutrient bioaccessibility. (**C**) Colonic targeting via mucoadhesion and microbiota-triggered enzymatic degradation enabling localized and site-specific delivery.

Release from food-grade microgels is not a passive diffusion event but the result of deliberate structural and chemical programming. These ingestible carriers are rationally engineered to respond to the evolving physicochemical conditions of the gastrointestinal (GI) tract, thereby enabling sequential protection and region-specific liberation of sensitive cargos—including probiotics, lipophilic vitamins, polyphenols, and essential mineral ions. Ideally, such systems are designed so that encapsulated bioactives (i) remain protected during gastric transit, (ii) avoid premature leakage in the upper small intestine, and (iii) are released only when and where they can exert maximal biological efficacy [61]. In this context, microgels function as programmable depots that dynamically negotiate the heterogeneous GI microenvironment—ranging from the acidic gastric lumen to the enzyme- and bile-rich small intestine and the microbially active colon—exhibiting region-specific release behavior (Figure 8) [100]. This spatiotemporal control arises from three interrelated mechanisms: (i) diffusion-limited retention under gastric conditions, (ii) swelling- and erosion-mediated matrix relaxation in the small intestine, and (iii) microbiota-triggered degradation coupled with mucoadhesive localization in the colon.

**Figure 9:**
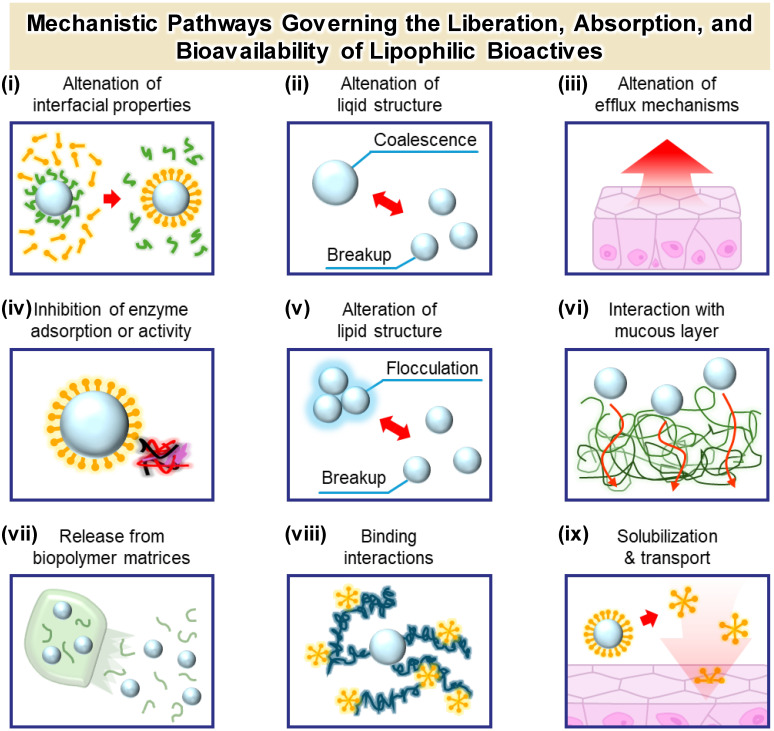
Mechanistic pathways governing the liberation, absorption, and bioavailability of lipophilic bioactives during gastrointestinal digestion. The schematic integrates nine interconnected processes that collectively determine the fate of lipophilic nutraceuticals: (**i**) release from the food matrix, (**ii**) particle size reduction and interfacial restructuring, (**iii**) epithelial transport across the intestinal barrier, (**iv**) protection against chemical degradation, (**v**) micelle formation and solubilization, (**vi**) diffusion within biopolymeric networks, (**vii**) enzymatic digestion and matrix disintegration, (**viii**) molecular interactions with surrounding macromolecules, and (**ix**) cellular uptake and systemic distribution. Together, these sequential and overlapping events define bioaccessibility, absorption efficiency, and ultimate bioavailability.

The first layer of control is diffusion-mediated retention within the microgel matrix (Figure 9i,vi). Within tightly crosslinked networks, attractive interactions—such as electrostatic binding, ionic coordination, or hydrophobic association—between the encapsulated cargo and the polymer scaffold markedly suppress molecular mobility and outward transport [105]. This diffusion-limited confinement is particularly critical for gastric protection of probiotics, where live bacteria embedded within dense Ca^2+^–alginate matrices are shielded from acid penetration during gastric residence [148,149,150]. Similarly, mineral ions (e.g., Fe^2+^/Fe^3+^) can be transiently coordinated within hydrated polymer cages, minimizing gastric irritation while enabling gradual intestinal liberation. In this context, release from the matrix represents the initial step governing the subsequent bioaccessibility cascade.

The second regulatory layer involves swelling- and erosion-driven structural transitions that occur upon exposure to intestinal conditions (Figure 9ii,v,vii). When a microgel swells in response to pH elevation or ionic shifts, increased hydration enlarges the network mesh size and accelerates outward flux [151]. Concurrently, bile salts and pancreatic lipases emulsify and digest internal lipid domains, leading to micelle formation and solubilization of lipophilic micronutrients such as carotenoids [147]. The interplay between matrix erosion, particle size reduction, and interfacial restructuring critically determines the efficiency of mixed micelle formation, which in turn governs epithelial uptake and systemic availability. Monodisperse microgels may additionally be engineered for temporally controlled disassembly to engage intestinal feedback pathways, including the “intestinal brake,” thereby influencing satiety and metabolic regulation [152].

The third layer consists of stimulus-responsive and mucoadhesive mechanisms that modulate site-specific release and epithelial interaction (Figure 9iii,viii,ix). pH-responsive shells (e.g., alginate–chitosan complexes) remain compact under gastric acidity but undergo swelling or delamination at near-neutral intestinal pH, enabling targeted delivery [70]. Functionalization with thiolated or cationic polysaccharides enhances mucoadhesion through electrostatic interactions and chain interpenetration with mucins, effectively anchoring the carrier to target tissues such as the inflamed colon and prolonging local residence time [86,153]. These interactions not only influence diffusion but also affect epithelial transport pathways and cellular uptake kinetics.

In practice, these mechanistic layers operate sequentially and synergistically, as illustrated in Figure 9. Release from the food matrix, micellar solubilization, diffusion through biopolymeric networks, enzymatic degradation, epithelial absorption, and systemic distribution are interconnected processes that collectively determine bioaccessibility and ultimate bioavailability. This integrated spatiotemporal programming underpins the emerging paradigm of “precision nutrition,” wherein bioactives are delivered with pharmaceutical-style targeting to modulate chronic disorders—including age-related macular degeneration—via mechanisms such as the gut–eye axis [94].

## 7. Applications of Food-Grade Microgel for AMD

**Figure 10:**
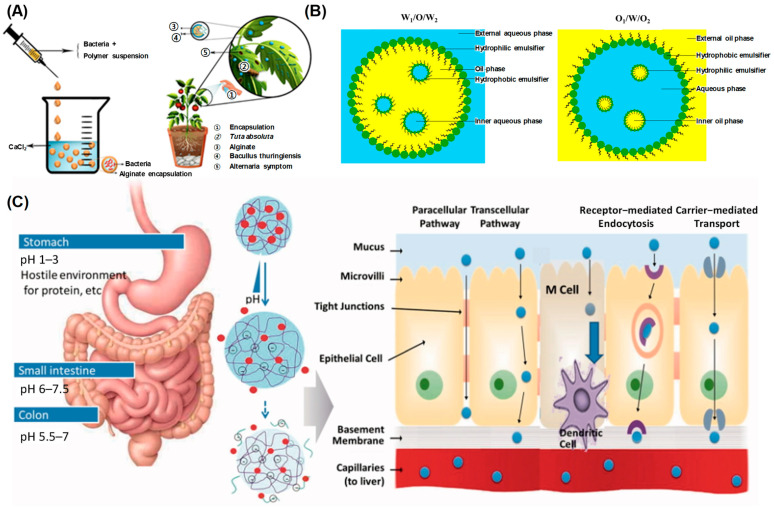
Food-grade microgel platforms for stabilization, co-encapsulation, and gastrointestinal-responsive delivery of AMD-related bioactives. (**A**) Alginate-based encapsulation strategy in which bioactives are incorporated into hydrated polymer networks through ionotropic gelation, forming microgels that physically entrap and protect sensitive compounds from oxidative and environmental degradation. (Adapted from Ref. [154] Copyright © 2021 MDPI) (**B**) Representative co-encapsulation architectures (O/W, W/O, W/O/W, O/W/O) enabling the simultaneous incorporation of hydrophilic and lipophilic bioactives within structured microgel systems to minimize antagonistic interactions and coordinate release profiles. (Adapted from Ref. [155] Copyright © 2024 MDPI) (**C**) Microgel-mediated gastrointestinal protection and region-specific release behavior, illustrating stability under acidic gastric conditions and swelling- or degradation-triggered release in the intestine to enhance systemic bioavailability. (Adapted from Ref. [156]. Copyright © 2010 Taylor & Francis).

As illustrated in Figure 10, food-grade microgels can be engineered to protect sensitive bioactives, enhance gastrointestinal stability and absorption, and enable multi-nutrient delivery strategies relevant to AMD.

### 7.1. Enhancement of Nutrient Stability for Retinal Protection

Many bioactive compounds utilized to mitigate AMD, including carotenoids, polyphenols, and omega-3 fatty acids, exhibit significant instability during processing, storage, and gastrointestinal transit [41,157]. These compounds are highly susceptible to oxidation, heat, and photodegradation, compromising their effective delivery to retinal tissues and limiting their biological activity [158]. Food-grade microgels provide a protective matrix that isolates these nutrients from deleterious environmental conditions, thereby preserving their structural integrity and bioactivity [159]. As illustrated in Figure 10A, alginate-based microgels are commonly fabricated via ionotropic gelation, in which bioactives are dispersed within an alginate precursor solution and subsequently crosslinked by divalent cations (e.g., Ca^2+^) to form a three-dimensional hydrogel network. The resulting Ca^2+^–alginate matrix creates a diffusion-limited polymer cage that physically entraps sensitive compounds within a hydrated, biopolymer-rich environment. This network architecture restricts oxygen permeation, reduces radical propagation, and enhances resistance to oxidative, thermal, and photochemical degradation [154]. Beyond alginate systems, other polysaccharide- and protein-based microgels—including those derived from pectin, gelatin, or whey protein—exhibit similar protective mechanisms by forming crosslinked matrices that buffer environmental fluctuations and stabilize encapsulated bioactives [105,160].

The stability enhancement conferred by microgel encapsulation is particularly critical for AMD-related nutrients such as lutein, zeaxanthin, resveratrol, curcumin, and DHA, which are inherently prone to degradation under physiological conditions [145,161]. By maintaining these compounds within a stabilized microenvironment and limiting pre-absorptive loss, microgels preserve functional potency. Moreover, the hydrated network structure enables subsequent controlled release in the intestinal environment and can be further engineered for mucoadhesive retention, supporting sustained systemic delivery of antioxidant and neuroprotective agents to the retina [55]. Such preservation of bioactivity is essential for attenuating oxidative stress, a principal driver RPE degeneration and AMD progression [8].

### 7.2. Improving Bioavailability and Targeted Uptake of Bioactives

Many AMD-related nutrients exhibit inherently low oral bioavailability due to poor aqueous solubility, limited dispersibility in the gastrointestinal (GI) tract, and rapid metabolic transformation. Carotenoids (e.g., lutein and zeaxanthin) and long-chain omega-3 fatty acids (e.g., DHA) are highly lipophilic and therefore inefficiently absorbed when administered in native crystalline or bulk oil forms [157].

Microgel-based co-encapsulation strategies address these limitations by organizing bioactives within compartmentalized colloidal architectures that enhance dispersion and controlled solubilization. As illustrated in Figure 10B, structured systems—including oil-in-water (O/W), water-in-oil (W/O), and multiple emulsion-derived configurations (W/O/W, O/W/O)—spatially segregate hydrophilic and lipophilic compounds into distinct internal domains [108,155]. This internal phase organization prevents direct antagonistic interactions between incompatible nutrients while maintaining structural integrity during GI transit. Within the intestinal lumen, the lipid-containing domains undergo enzymatic digestion by pancreatic lipases in the presence of bile salts, generating monoacylglycerols and free fatty acids that assemble into mixed micelles [162]. The microgel-templated dispersion state enhances interfacial accessibility, accelerates lipid hydrolysis, and promotes efficient micellar incorporation of lipophilic micronutrients. This process facilitates epithelial uptake and subsequent chylomicron-mediated systemic transport, thereby significantly improving apparent bioavailability and supporting effective retinal delivery of AMD-relevant compounds.

Furthermore, co-encapsulation architectures can be integrated with pH-sensitive or enzyme-responsive microgel matrices to synchronize release kinetics with optimal intestinal absorption windows [156]. By coupling spatial compartmentalization with stimuli-responsive matrix relaxation, these systems reduce premature degradation, maintain nutrient stability during gastric transit, and enable coordinated bioactive liberation in the small intestine. This precision-delivery strategy is conceptually aligned with landmark clinical interventions such as AREDS and AREDS2, which emphasize the necessity of maintaining adequate systemic concentrations of carotenoids, antioxidants, and essential fatty acids for effective AMD management [43,163].

### 7.3. Microgel-Based Encapsulation for Targeted Delivery

Beyond enhancing stability and systemic absorption, microgel systems can be rationally engineered to achieve region-specific gastrointestinal (GI) release of AMD-relevant nutrients. The physicochemical properties of microgels—including particle size, surface charge, hydrophobicity, and cross-link density—can be precisely tuned to modulate matrix permeability and environmentally responsive disassembly behavior [55]. As illustrated in Figure 10C [156], structured microgel platforms remain compact and diffusion-restricted under acidic gastric conditions (pH 1–3), thereby limiting premature cargo leakage and protecting labile bioactives from acid-induced degradation. Upon transition to the small intestine (pH ~6–7.5), changes in ionic strength, pH, and exposure to bile salts and digestive enzymes induce network swelling, partial erosion, or matrix relaxation. This environmentally triggered transformation enables spatially regulated payload liberation at physiologically optimal absorption sites. For instance, pH-responsive microgels shield nutrients during gastric transit and initiate release only after exposure to neutral or mildly alkaline intestinal environments [156]. Similarly, enzyme-degradable systems fabricated from natural biopolymers such as gelatin or starch derivatives undergo controlled matrix disintegration in concert with digestive activity, synchronizing nutrient release with lipid digestion and micelle formation processes [105]. In the context of Figure 10C, this staged GI-responsive behavior enhances bioaccessibility while minimizing luminal loss and oxidative degradation of sensitive compounds [55,156].

Although nutrient delivery for AMD primarily depends on systemic absorption following oral intake, the conceptual framework depicted in Figure 10C highlights how controlled gastrointestinal release contributes to sustained systemic exposure. Stable micellar incorporation and efficient epithelial transport ultimately support chylomicron-mediated distribution of carotenoids, polyphenols, and omega-3 fatty acids, facilitating their accumulation in retinal tissues [54]. By maintaining consistent circulating levels of antioxidant and anti-inflammatory nutrients, microgel-enabled delivery platforms may indirectly modulate key pathogenic mechanisms underlying AMD, including oxidative stress, chronic inflammation, and mitochondrial dysfunction within the RPE. The protective and GI-responsive functionalities shown in Figure 10C position food-grade microgels as a strategic platform for next-generation nutritional or medical food interventions aimed at improving the precision, reliability, and therapeutic relevance of AMD-targeted nutrient delivery.

### 7.4. Microgel-Based Multi-Nutrient Synergy Platforms

AMD management increasingly emphasizes the use of multi-nutrient formulations, as robustly demonstrated by clinical trials like AREDS2, which combine carotenoids, antioxidant vitamins, zinc, and omega-3 fatty acids to achieve synergistic protective effects [43]. However, the co-formulation of these diverse compounds presents significant chemical and technical challenges: nutrient-nutrient interactions often lead to chemical incompatibility, reduced stability (e.g., between Ascorbic acid and metallic ions), or diminished overall bioavailability [158,164].

Microgels offer a highly structured environment that can effectively minimize antagonistic interactions. They can either segregate individual components within a single matrix or co-encapsulate them in a spatially organized manner, thereby preserving the intrinsic bioactivity of each nutrient [55]. Furthermore, these designs enable coordinated release profiles that are optimized for combined efficacy in the gastrointestinal tract.

The ability of microgels to incorporate both hydrophilic (e.g., vitamins and polyphenols) and lipophilic (e.g., carotenoids and omega-3 fatty acids) compounds further supports the development of sophisticated multi-nutrient synergy platforms tailored for AMD prevention [108]. For instance, lipophilic components may be co-delivered within lipid-enriched domains, while hydrophilic molecules can be embedded within the surrounding hydrophilic polymer network. This structural compartmentalization enhances antioxidant capacity and stability [146]. This precision engineering approach aligns with the broader trend toward precision nutrition and functional food innovation, providing a scientifically grounded pathway to maximize the clinical relevance of nutrient-based strategies for long-term retinal health.

## 8. Conclusions and Future Directions

We have comprehensively evaluated the emergence of food-grade microgel systems as a sophisticated and clinically relevant platform for the targeted delivery of nutrients essential to managing AMD. Our analysis confirms that while nutritional interventions—specifically those involving carotenoids, omega-3 fatty acids, and antioxidant vitamins—are critical for delaying disease progression, their biological efficacy is fundamentally constrained by poor gastrointestinal stability and low aqueous solubility. We have detailed how microgels, engineered from biocompatible biopolymer networks, resolve these limitations by functioning as programmable reservoirs that shield sensitive bioactives from oxidative, thermal, and photochemical degradation. By modulating the physicochemical properties of these carriers, it is possible to achieve precise spatiotemporal release at optimized absorption sites, thereby maximizing systemic bioavailability and supporting the critical gut-retina axis.

Despite the significant achievements in laboratory-scale innovation, several technical and regulatory hurdles must be addressed to fully realize the potential of microgel-based precision nutrition. The following sections outline the key emerging issues and future research directions required to bridge the gap between material design and clinical application. The integration of food-grade microgels into the clinical management of AMD represents a paradigm shift in precision nutrition. However, bridging the gap between innovative laboratory prototypes and consumer-ready functional foods requires overcoming significant hurdles in safety, engineering, and functional intelligence.

### 8.1. Fabrication Scalability, Regulatory Pathways, and Commercial Translation

The transition from high-precision laboratory prototypes to industrial-scale functional foods requires a dual focus on engineering and safety. While microfluidic techniques offer unparalleled control over monodispersity, their current throughput remains a bottleneck for mass-market AMD interventions. For the large-scale delivery of minerals and cofactors, mechanical atomization processes such as spray drying and centrifugal extrusion offer the necessary scalability to produce tons of fortified powders annually.

However, the scalability of probiotics presents unique challenges, as thermal exposure during conventional spray drying can lead to significant loss of viability. To address this, the industry is shifting toward ‘cold-processing’ mechanical alternatives—including electrospraying and spray chilling—which preserve the metabolic activity of live cells at scale. Regardless of the fabrication route, the use of GRAS-certified biopolymers (e.g., alginate, pectin, and globular proteins) is paramount. Explicitly utilizing these ingredients ensures that the resulting microgels meet the stringent safety standards of the gut-eye axis therapeutic framework, facilitating consumer trust and regulatory clearance for clinical use.

Beyond material safety, the transition from laboratory prototypes to market-ready products is governed by complex international regulatory frameworks. Despite the promising potential of food-grade microgels as delivery platforms for nutraceuticals targeting AMD, their large-scale commercialization requires careful consideration of regulatory approval pathways and industrial feasibility [165]. In the United States, ingredients incorporated into food products must generally comply with the GRAS framework administered by the U.S. Food and Drug Administration (FDA) [166]. Many biopolymers commonly used in microgel systems—including proteins (e.g., whey protein, casein, and gelatin) and polysaccharides (e.g., alginate, pectin, and chitosan)—already possess GRAS status or are widely accepted as food additives [123]. However, regulatory evaluation may still be required when these materials are used in novel structural formats or delivery systems, particularly when particle size, physicochemical properties, or intended functionality differ substantially from traditional food applications [167]. In the European Union, regulatory oversight is primarily governed by the European Food Safety Authority (EFSA) under the Novel Food Regulation (EU) 2015/2283. Materials that have not been consumed to a significant degree prior to May 1997 may require formal safety assessments before commercialization. Although many food-grade polymers used in microgels are individually approved food ingredients, the introduction of engineered colloidal structures or nano/micro-scale delivery systems may trigger additional safety evaluations regarding digestion behavior, bioavailability, and long-term exposure [167,168]. From a commercial perspective, several practical challenges also influence industrial translation. These include scalability of production technologies, cost of encapsulation materials, regulatory documentation requirements, and the need for standardized characterization methods to ensure batch-to-batch consistency. In addition, consumer perception of engineered food delivery systems may influence market adoption, particularly when products involve engineered colloidal structures, which require transparent labeling and clear communication of safety benefits to ensure public acceptance. Addressing these regulatory and commercial considerations will therefore be essential for translating microgel-based nutraceutical delivery systems from laboratory-scale research to clinically relevant dietary interventions for AMD [169].

### 8.2. Advanced Architectures for Multi-Nutrient Synergy

Future research should explore more complex, heterogeneous interface designs that leverage tailored biopolymer interactions. By constructing multilayered or hybrid networks, researchers can effectively segregate individual components to prevent antagonistic interactions while promoting coordinated release. For example, co-encapsulating lipophilic carotenoids within lipid-rich domains and hydrophilic vitamins within the surrounding hydrogel matrix allows for the development of sophisticated synergy platforms. Such architectures can be optimized to deliver pharmaceutical-style precision in a fully food-grade format, ensuring that multi-nutrient payloads are released in a sequence that maximizes their collective bioactivity.

### 8.3. Computational Modeling of Digestive Dynamics

To unravel the complex interfacial phenomena occurring within the gastrointestinal tract, the integration of advanced computational modeling is essential. Electrodynamic and fluid-dynamic simulations can predict local pH-responsive swelling, matrix erosion, and nutrient diffusion under varying physiological conditions. Mathematical models that incorporate digestive kinetics and intestinal transit factors will provide quantitative parameters for optimizing microgel design. Future work should aim to integrate real-time feedback models and time-domain simulations to achieve a predictive, rather than reactive, approach to delivery system engineering.

### 8.4. AI-Driven Material Exploration and Precision Nutrition

The integration of novel biopolymers with artificial intelligence (AI) represents a transformative strategy for accelerating the discovery of optimal microgel formulations. AI-driven technologies can efficiently explore vast material design spaces to identify biopolymer combinations with superior encapsulation efficiency and environmental stability. Machine learning models trained on digestive data sets can predict release characteristics and bioaccessibility, significantly shortening the development cycle for personalized nutrition. However, the current effectiveness of these models is constrained by the absence of high-quality, standardized in vivo data sets. To achieve true predictive intelligence, future research must prioritize the collection of real-world biological performance metrics to bridge the gap between in vitro simulations and actual metabolic outcomes.

### 8.5. Adaptive and “Smart” Delivery Platforms

Incorporating adaptive functionalities into microgel interfaces is a defining frontier for next-generation nutraceuticals. We envision the development of stimuli-responsive carriers that can autonomously adjust their delivery rates in response to external cues or internal physiological shifts. This includes the integration of multi-trigger release mechanisms—where release is synergistically activated by pH transitions and microbial enzymatic activity—as well as the potential for real-time monitoring tags. These “smart” platforms will enable real-time performance modulation, ensuring that nutrient delivery is precisely tuned to the body’s dynamic needs, thereby offering a robust and proactive route to preserving long-term visual health.

### 8.6. Bridging the Gap: In Vivo Limitations and Proposed Validation

Despite the significant achievements in laboratory-scale innovation, a critical bottleneck in the clinical translation of food-grade microgels is the scarcity of in vivo data. Most existing studies are performed within in vitro simulated gastrointestinal environments, which cannot fully replicate the complex physiological variables of the human body, such as peristaltic shear, gastric emptying dynamics, and the significant inter-individual variability of the gut microbiota. These factors are essential for determining the actual efficacy of the gut-eye axis pathway. To overcome these limitations, we propose a systematic validation framework consisting of three primary stages:

- Spatiotemporal Tracking: Utilizing near-infrared (NIR) or fluorescence imaging in rodent models to track the movement and integrity of microgels through the GI tract, ensuring they resist gastric acid and release cargo specifically in the absorption-rich small intestine.
- Retinal Efficacy Profiling: Measuring the accumulation of delivered xanthophylls (lutein/zeaxanthin) and the preservation of RPE health in established AMD animal models, such as light-damage or aging-accelerated mice.
- Clinical Bioavailability Trials: Conducting human pilot studies to compare the systemic bioavailability and safety of microgel-based delivery versus standard crystalline or oil-based supplements used in the AREDS2 regimens.

By implementing these validation strategies, researchers can confirm that the structural protections provided by food-grade microgels translate into measurable neuroprotective benefits for the retina.

### 8.7. Clinical Translation and AMD Patient Trials

Future research should further explore the clinical translation of microgel-based nutraceutical delivery systems for AMD through well-designed human intervention studies. In particular, randomized, double-blind, placebo-controlled trials evaluating microgel-encapsulated carotenoids, polyphenols, or other retinal-protective bioactives could provide valuable evidence regarding improvements in systemic absorption and retinal bioavailability compared with conventional nutraceutical formulations [43,170,171]. In such studies, macular pigment optical density (MPOD) may serve as a clinically relevant biomarker reflecting retinal carotenoid accumulation and macular nutritional status, which can be assessed using techniques such as heterochromatic flicker photometry or fundus autofluorescence imaging [172]. Longitudinal monitoring over approximately 6–12 months could further enable evaluation of MPOD changes, circulating carotenoid concentrations, and visual function outcomes in AMD patients receiving microgel-enhanced nutraceutical interventions [43,172]. Such translational studies would help bridge the gap between food colloid engineering and clinical nutrition, providing critical evidence supporting microgel delivery systems as promising next-generation dietary strategies for AMD prevention and management.

## Figures and Tables

**Table 1:** Key nutrients for AMD management: Mechanisms, limitations and the role of microgel delivery systems.

Category	Nutrients	Therapeutic Mechanisms in AMD	Physicochemical and Bioavailability Challenges	Advantages of Microgel Encapsulation
Antioxidant Vitamins	Vitamin C, Vitamin E, Vitamin A derivatives	Scavenging reactive oxygen species; Supporting photoreceptor maintenance	Chemical instability; Degradation under environmental stress (light/heat)	Protection against degradation; Enhanced stability during storage
Carotenoids	Lutein, Zeaxanthin, Meso-zeaxanthin	Blue-light filtering; Potent antioxidant activity concentrated in the macula	Low water solubility; Poor intestinal absorption; Susceptibility to oxidation and light	Enhanced water dispersibility; Improved bioavailability; Protection from light-induced degradation
Omega-3 Fatty Acids	DHA, EPA	Anti-inflammatory actions; Maintaining photoreceptor structural integrity and retinal homeostasis	High susceptibility to oxidation (rancidity); Very low water solubility; Limited uptake	Prevention of oxidation; Controlled release kinetics; Masking of off-flavors (implied)
Polyphenols and Minerals	Resveratrol, Anthocyanins, Curcumin, Zinc	Modulating oxidative and inflammatory pathways; Complementary RPE protection	Rapid metabolism; Poor systemic bioavailability; Chemical instability in physiological fluids	Targeted delivery to retinal tissues; Modulation of release profile to match physiological demands

**Table 2:** Recent clinical breakthroughs and bioavailability enhancement technologies (2024–2026).

Intervention	Target Population	Key Clinical Findings	Bioavailability Technology	Ref.
AREDS2 (L/Z 10:2 mg)	Geographic Atrophy patients	55% reduction in GA progression toward fovea (Foveal Sparing).	Micellar Solubilization: Optimizes pigment density in the macula.	[49]
Lutein/Zeaxanthin	High digital screen users	Significant improvement in digital eye strain and sleep quality.	Oil-based Nanoemulsion: Increases rapid absorption for immediate relief.	[50]
Polyphenols (Nano-form)	Oxidative Stress Models	10-fold increase in ROS scavenging efficacy in retinal models.	Chitosan Nanoparticles: Enhances ocular surface retention and bioavailability.	[51]
L/Z + Anthocyanins	Retinal Degeneration	Synergistic RPE protection and prevention of light-induced apoptosis.	SNEDDS: Oral bioavailability increased by over 200%.	[52]
Carotenoids and Flavonoids	Healthy Adults	Established the role of lipid-phase interactions in maximizing pigment uptake.	Self-assembling Micelles: Facilitates intestinal lymphatic transport.	[53]

**Table 3:** Comparison of commonly used polysaccharide carriers for microgel-based nutraceutical delivery systems.

Polysaccharide	Gelation Mechanism	Advantages (Specific to AMD Delivery)	Limitations	Typical Applications
Alginate	Ionic crosslinking with Ca^2+^	Mild gelation; High encapsulation efficiency; Superior protection for heat-sensitive carotenoids (Lutein/Zeaxanthin)	Poor stability in high ionic strength environments; Potential for premature burst release	Encapsulation of vitamins, carotenoids, and probiotics
Chitosan	Ionic crosslinking/Polyelectrolyte complexation	Strong mucoadhesion; pH-responsive release; Enhances retention on ocular surface and intestinal mucosa	Limited solubility at neutral pH; Requires acidic conditions for initial processing	Controlled intestinal delivery; Ocular surface nutrient delivery
Pectin	Calcium-mediated gelation	Biodegradable; Microbiota-triggered degradation; Ideal for colon-targeted gut-eye axis modulation	Relatively weak mechanical strength compared to alginate	Colon-specific delivery of polyphenols and antioxidants
Carrageenan	Thermoreversible gelation with cations	Strong gel structure; Excellent mechanical stability; High water retention for shelf-life extension	Sensitivity to specific ionic conditions (e.g., K^+^); Potential regulatory constraints in some regions	Stabilization of multi-nutrient systems; Texture modification in functional foods

**Table 4:** Comparative analysis of physicochemical and mechanical fabrication methods for food-grade microgels.

Method Category	Primary Examples	Industrial Advantages	Technical Limitations	Suitability for AMD Bioactives
Physicochemical	Complex coacervation, Ionic gelation	Mild conditions; Preserves heat-sensitive bioactives	Limited control over monodispersity; Batch variability	Highest; Ideal for heat-sensitive Lutein, Zeaxanthin, and Probiotics
Mechanical	Spray drying, Microfluidics, Homogenization	High scalability; Precise size control (1–100 μm)	Potential shear/thermal stress; Oxidative risk for DHA/EPA	High; Best for large-scale production of fortified powders

## Data Availability

The data presented in this study are available on request from the corresponding author.

## References

[1] Thomas C.J., Mirza R.G., Gill M.K. (2021). Age-Related Macular Degeneration. Med. Clin. North. Am..

[2] Mitchell P., Wang J.J., Smith W., Leeder S.R. (2002). Smoking and the 5-year incidence of age-related maculopathy: The Blue Mountains Eye Study. Arch. Ophthalmol..

[3] Chong E.W., Kreis A.J., Wong T.Y., Simpson J.A., Guymer R.H. (2008). Alcohol consumption and the risk of age-related macular degeneration: A systematic review and meta-analysis. Am. J. Ophthalmol..

[4] Chakravarthy U., Wong T.Y., Fletcher A., Piault E., Evans C., Zlateva G., Buggage R., Pleil A., Mitchell P. (2010). Clinical risk factors for age-related macular degeneration: A systematic review and meta-analysis. BMC Ophthalmol..

[5] Babaker R., Alzimami L., Al Ameer A., Almutairi M., Alam Aldeen R., Alshatti H., Al-Johani N., Al Taisan A. (2025). Risk factors for age-related macular degeneration: Updated systematic review and meta-analysis. Medicine.

[6] Guymer R.H., Campbell T.G. (2023). Age-related macular degeneration. Lancet.

[7] de Jong S., Tang J., Clark S.J. (2023). Age-related macular degeneration: A disease of extracellular complement amplification. Immunol. Rev..

[8] Beatty S., Koh H., Phil M., Henson D., Boulton M. (2000). The role of oxidative stress in the pathogenesis of age-related macular degeneration. Surv. Ophthalmol..

[9] Borodi P.G., Slevin M. (2025). Exploring the role of inflammation in age-related macular degeneration: New insights and implications for future therapies. Rom. J. Morphol. Embryol..

[10] Apte R.S., Chen D.S., Ferrara N. (2019). VEGF in Signaling and Disease: Beyond Discovery and Development. Cell.

[11] Ferris F.L., Wilkinson C.P., Bird A., Chakravarthy U., Chew E., Csaky K., Sadda S.R. (2013). Beckman Initiative for Macular Research Classification Commitee. Clinical classification of age-related macular degeneration. Ophthalmology.

[12] Kulkarni A.D., Kuppermann B.D. (2005). Wet age-related macular degeneration. Adv. Drug Deliv. Rev..

[13] Raddatz L., Lavrentieva A., Pepelanova I., Bahnemann J., Geier D., Becker T., Scheper T., Beutel S. (2018). Development and Application of an Additively Manufactured Calcium Chloride Nebulizer for Alginate 3D-Bioprinting Purposes. J. Funct. Biomater..

[14] Wong W.L., Su X., Li X., Cheung C.M., Klein R., Cheng C.Y., Wong T.Y. (2014). Global prevalence of age-related macular degeneration and disease burden projection for 2020 and 2040: A systematic review and meta-analysis. Lancet Glob. Health.

[15] Jeong Y.D., Park S., Kim M.S., Hong S.H., Aalruz H., Abate Y.H., Abbasgholizadeh R., Abd El Hafeez S., Abdullahi A., Aboagye R.G. (2025). Global burden of vision impairment due to age-related macular degeneration, 1990–2021, with forecasts to 2050: A systematic analysis for the Global Burden of Disease Study 2021. Lancet Glob. Health.

[16] Kim M.S., Nam S., Park S.J., Lee J., Woo S.J. (2025). 10-Year Change and Projection in Prevalence and Incidence of Exudative Age-Related Macular Degeneration in Korea. J. Korean Med. Sci..

[17] Cheung C.M.G., Lai T.Y.Y., Teo K., Ruamviboonsuk P., Chen S.-J., Kim J.E., Gomi F., Koh A.H., Kokame G., Jordan-Yu J.M. (2021). Polypoidal Choroidal Vasculopathy: Consensus Nomenclature and Non-Indocyanine Green Angiograph Diagnostic Criteria from the Asia-Pacific Ocular Imaging Society PCV Workgroup. Ophthalmology.

[18] Stahl A. (2020). The Diagnosis and Treatment of Age-Related Macular Degeneration. Dtsch. Arztebl. Int..

[19] Shirian J.D., Shukla P., Singh R.P. (2025). Exploring new horizons in neovascular age-related macular degeneration: Novel mechanisms of action and future therapeutic avenues. Eye.

[20] Lin P. (2018). The role of the intestinal microbiome in ocular inflammatory disease. Curr. Opin. Ophthalmol..

[21] Andriessen E.M.M.A., Wilson A.M., Mawambo G., Dejda A., Miloudi K., Sennlaub F., Sapieha P. (2016). Gut microbiota influences pathological angiogenesis in obesity-driven choroidal neovascularization. EMBO Mol. Med..

[22] Zinkernagel M.S., Zysset-Burri D.C., Keller I., Berger L.E., Leichtle A.B., Largiadèr C.R., Fiedler G.M., Wolf S. (2017). Association of the Intestinal Microbiome with the Development of Neovascular Age-Related Macular Degeneration. Sci. Rep..

[23] Beirao S., Pereira P.M.R., Fernandes R., Tome J.P.C. (2025). Photosensitizer formulations in photodynamic therapy of age-related macular degeneration. Eur. J. Med. Chem..

[24] Schmidt-Erfurth U.M., Richard G., Augustin A., Aylward W.G., Bandello F., Corcostegui B., Cunha-Vaz J., Gaudric A., Leys A., Schlingemann R.O. (2007). Guidance for the treatment of neovascular age-related macular degeneration. Acta Ophthalmol. Scand..

[25] Heier J.S., Khanani A.M., Quezada Ruiz C., Basu K., Ferrone P.J., Brittain C., Figueroa M.S., Lin H., Holz F.G., Patel V. (2022). Efficacy, durability, and safety of intravitreal faricimab up to every 16 weeks for neovascular age-related macular degeneration (TENAYA and LUCERNE): Two randomised, double-masked, phase 3, non-inferiority trials. Lancet.

[26] Investigators P., Korobelnik J.-F., Lanzetta P., Leal S., Holz F.G., Clark W.L., Eichenbaum D., Iida T., Sun X., Berliner A.J. (2026). Intravitreal Aflibercept 8 mg in Neovascular Age-Related Macular Degeneration: Ninety-Six-Week Results from the Randomized Phase 3 PULSAR Trial. Ophthalmology.

[27] Heier J.S., Lad E.M., Holz F.G., Rosenfeld P.J., Guymer R.H., Boyer D., Grossi F., Baumal C.R., Korobelnik J.-F., Slakter J.S. (2023). Pegcetacoplan for the treatment of geographic atrophy secondary to age-related macular degeneration (OAKS and DERBY): Two multicentre, randomised, double-masked, sham-controlled, phase 3 trials. Lancet.

[28] Investigators G.T., Khanani A.M., Danzig C.J., Heier J.S., Jaffe G.J., Kaiser P.K., Lally D.R., Patel S.S., Vajzovic L., Weng C.Y. (2025). Avacincaptad Pegol for Geographic Atrophy Secondary to Age-Related Macular Degeneration: Two-Year Efficacy and Safety Results from the GATHER2 Phase 3 Trial. Ophthalmology.

[29] Sarkar A., Jayesh Sodha S., Junnuthula V., Kolimi P., Dyawanapelly S. (2022). Novel and investigational therapies for wet and dry age-related macular degeneration. Drug Discov. Today.

[30] Wang Z., Zhang Y., Xu C., Peng A., Qin H., Yao K. (2025). Advancements in age-related macular degeneration treatment: From traditional anti-VEGF to emerging therapies in gene, stem cell, and nanotechnology. Biochem. Pharmacol..

[31] Singh R.P., Avery R.L., Barakat M.R., Kim J.E., Kiss S. (2024). Evidence-Based Use of Bevacizumab in the Management of Neovascular Age-Related Macular Degeneration. Ophthalmic Surg. Lasers Imaging Retin..

[32] Su Y., Wu J., Gu Y. (2018). Photodynamic therapy in combination with ranibizumab versus ranibizumab monotherapy for wet age-related macular degeneration: A systematic review and meta-analysis. Photodiagn. Photodyn. Ther..

[33] Ashraf M., Souka A.A.R. (2017). Aflibercept in age-related macular degeneration: Evaluating its role as a primary therapeutic option. Eye.

[34] Radke N.V., Mohamed S., Brown R.B., Ibrahim I., Chhablani J., Amin S.V., Tsang C.W., Brelen M.E., Raichand N.S., Fang D. (2023). Review on the Safety and Efficacy of Brolucizumab for Neovascular Age-Related Macular Degeneration From Major Studies and Real-World Data. Asia Pac. J. Ophthalmol..

[35] Krebs I., Glittenberg C., Ansari-Shahrezaei S., Hagen S., Steiner I., Binder S. (2013). Non-responders to treatment with antagonists of vascular endothelial growth factor in age-related macular degeneration. Br. J. Ophthalmol..

[36] Wykoff C.C., Clark W.L., Nielsen J.S., Brill J.V., Greene L.S., Heggen C.L. (2018). Optimizing Anti-VEGF Treatment Outcomes for Patients with Neovascular Age-Related Macular Degeneration. J. Manag. Care Spec. Pharm..

[37] Thomas C.N., Sim D.A., Lee W.H., Alfahad N., Dick A.D., Denniston A.K., Hill L.J. (2022). Emerging therapies and their delivery for treating age-related macular degeneration. Br. J. Pharmacol..

[38] Rodriguez-Amaya D.B. (2015). Carotenoids as food colorants and precursors of aroma compounds. Food Carotenoids.

[39] Armulik A., Genové G., Betsholtz C. (2011). Pericytes: Developmental, Physiological, and Pathological Perspectives, Problems, and Promises. Dev. Cell.

[40] Saint-Geniez M., Maharaj A.S.R., Walshe T.E., Tucker B.A., Sekiyama E., Kurihara T., Darland D.C., Young M.J., D’Amore P.A. (2008). Endogenous VEGF Is Required for Visual Function: Evidence for a Survival Role on Müller Cells and Photoreceptors. PLoS ONE.

[41] Bernstein P.S., Li B., Vachali P.P., Gorusupudi A., Shyam R., Henriksen B.S., Nolan J.M. (2016). Lutein, zeaxanthin, and meso-zeaxanthin: The basic and clinical science underlying carotenoid-based nutritional interventions against ocular disease. Prog. Retin. Eye Res..

[42] Age-Related Eye Disease Study Research Group (2001). A randomized, placebo-controlled, clinical trial of high-dose supplementation with vitamins C and E, beta carotene, and zinc for age-related macular degeneration and vision loss: AREDS report no. 8. Arch. Ophthalmol..

[43] Age-Related Eye Disease Study 2 Research G. (2013). Lutein + zeaxanthin and omega-3 fatty acids for age-related macular degeneration: The Age-Related Eye Disease Study 2 (AREDS2) randomized clinical trial. JAMA.

[44] SanGiovanni J.P., Chew E.Y. (2005). The role of omega-3 long-chain polyunsaturated fatty acids in health and disease of the retina. Prog. Retin. Eye Res..

[45] Bungau S., Abdel-Daim M.M., Tit D.M., Ghanem E., Sato S., Maruyama-Inoue M., Yamane S., Kadonosono K. (2019). Health Benefits of Polyphenols and Carotenoids in Age-Related Eye Diseases. Oxid. Med. Cell Longev..

[46] Rein M.J., Renouf M., Cruz-Hernandez C., Actis-Goretta L., Thakkar S.K., da Silva Pinto M. (2013). Bioavailability of bioactive food compounds: A challenging journey to bioefficacy. Br. J. Clin. Pharmacol..

[47] D’Archivio M., Filesi C., Vari R., Scazzocchio B., Masella R. (2010). Bioavailability of the polyphenols: Status and controversies. Int. J. Mol. Sci..

[48] de Souza Simoes L., Madalena D.A., Pinheiro A.C., Teixeira J.A., Vicente A.A., Ramos O.L. (2017). Micro- and nano bio-based delivery systems for food applications: In vitro behavior. Adv. Colloid. Interface Sci..

[49] Keenan T.D.L., Agrón E., Keane P.A., Domalpally A., Chew E.Y., Age-Related Eye Disease Study Research Group, Age-Related Eye Disease Study 2 Research Group (2025). Oral Antioxidant and Lutein/Zeaxanthin Supplements Slow Geographic Atrophy Progression to the Fovea in Age-Related Macular Degeneration. Ophthalmology.

[50] Lopresti A.L., Smith S.J. (2025). The effects of lutein/ zeaxanthin (Lute-gen^®^) on eye health, eye strain, sleep quality, and attention in high electronic screen users: A randomized, double-blind, placebo-controlled study. Front. Nutr..

[51] D’Angelo A., Giannaccare G., Lixi F., Troisi M., Adamo G.G., Lee D., De Pascale I., Pellegrino A., Vitiello L. (2026). Polyphenols and Eye Health: A Narrative Review of the Literature on the Therapeutic Effects for Ocular Diseases. Nutrients.

[52] Yoo J., Baskaran R., Yoo B.K. (2013). Self-nanoemulsifying drug delivery system of lutein: Physicochemical properties and effect on bioavailability of warfarin. Biomol. Ther..

[53] Desmarchelier C., Borel P. (2017). Overview of carotenoid bioavailability determinants: From dietary factors to host genetic variations. Trends Food Sci. Technol..

[54] Harrison E.H. (2019). Mechanisms of Transport and Delivery of Vitamin A and Carotenoids to the Retinal Pigment Epithelium. Mol. Nutr. Food Res..

[55] McClements D.J. (2017). Designing biopolymer microgels to encapsulate, protect and deliver bioactive components: Physicochemical aspects. Adv. Colloid. Interface Sci..

[56] Ahmadzadeh S., Barekat S., Ubeyitogullari A. (2025). Enhancing lutein and anthocyanins stability and bioaccessibility through simultaneous encapsulation using coaxial 3D food printing. npj Sci. Food.

[57] Briones S.C., Mussagy C.U., Farias F.O., Córdova A. (2025). Functional Hydrogels in Food Applications: A Review of Crosslinking Technologies, Encapsulation Trends, and Emerging Challenges. Polymers.

[58] Qu S., Bai H., Xu Y., Li B., Wang X. (2025). Nature’s sophisticated architecture: A strategic approach for protein-polysaccharide complexes to address the “shelf-life vs. bioavailability” dilemma of polyphenols in food applications. Front. Nutr..

[59] Machado M., Costa E.M., Silva S. (2025). Soft Gels in Food Systems: Recent Advances, Applications, and Technological Innovations. Gels.

[60] Zhang J., Liu B., Xie Q., Wang J., Ma L., Yang C., Yang X., Guo L., Su Y., Jiang Y. (2026). Polysaccharide-Based Microgels for Probiotic Delivery: Advances in Design, Fabrication, and Functional Food Applications. Trends Food Sci. Technol..

[61] Dong X., Wang Y., Chen X.D., Selomulya C. (2026). Designing Protein Gels for Nutrient Delivery: From Formulation to Digestion. Compr. Rev. Food Sci. Food Saf..

[62] Kwiecień I., Kwiecień M. (2018). Application of Polysaccharide-Based Hydrogels as Probiotic Delivery Systems. Gels.

[63] Milivojević M., Popović A., Pajić-Lijaković I., Šoštarić I., Kolašinac S., Stevanović Z.D. (2023). Alginate Gel-Based Carriers for Encapsulation of Carotenoids: On Challenges and Applications. Gels.

[64] Farjami T., Madadlou A. (2017). Fabrication methods of biopolymeric microgels and microgel-based hydrogels. Food Hydrocoll..

[65] Deng C., Liu Y., Li J., Yadav M.P., Yin L. (2018). Diverse rheological properties, mechanical characteristics and microstructures of corn fiber gum/soy protein isolate hydrogels prepared by laccase and heat treatment. Food Hydrocoll..

[66] Wang R., Zeng M.-Q., Wu Y.-W., Teng Y.-X., Wang L.-H., Li J., Xu F.-Y., Chen B.-R., Han Z., Zeng X.-A. (2023). Enhanced encapsulation of lutein using soy protein isolate nanoparticles prepared by pulsed electric field and pH shifting treatment. Food Chem..

[67] Zhang M., Liang B., He H., Ji C., Cui T., Sun C. (2021). Influence of Whey Protein Micro-Gel Particles and Whey Protein Micro-Gel Particles-Xanthan Gum Complexes on the Stability of O/W Emulsions. Polymers.

[68] Shahidi F., Dissanayaka C.S. (2023). Binding of carotenoids to proteins: A review. J. Food Bioact..

[69] Egan T., Jacquier J.-C., Rosenberg Y., Rosenberg M. (2013). Cold-set whey protein microgels for the stable immobilization of lipids. Food Hydrocoll..

[70] Zhu W., Xia M., He Y., Huang Q., Liao Z., Wang X., Zhou X., Duan X. (2026). Hydrogels as Promising Carriers for Ophthalmic Disease Treatment: A Comprehensive Review. Gels.

[71] Cai J., Zhang S., Liu S., Li X., Wan Z., Noskov B.A., Yang X. (2025). pH-responsive microgels constructed from soy protein coacervates: Structure and rheology at the oil-water interface. Food Hydrocoll..

[72] Rout S., Bakir S., Khanal S., Kaur G., Khan Z.S., Aijaz T., Sharma J., Bhat M.S., Mehraj F., Vahid G.S. (2025). Recent advances in plant protein microgels encapsulated with bioactive compounds. Food Hydrocoll. Health.

[73] Scopelliti G., Ferraro C., Parisi O.I., Dattilo M. (2026). Recent Developments in Protein-Based Hydrogels for Advanced Drug Delivery Applications. Pharmaceutics.

[74] Gomes A., Sobral P.J. (2022). Plant Protein-Based Delivery Systems: An Emerging Approach for Increasing the Efficacy of Lipophilic Bioactive Compounds. Molecules.

[75] Saif A., Anjum L., Faisal Z., Akram N., Shah Y.A., Irfan R., Saeed F., Afzaal M., Asif Shah M. (2023). Recent advances in protein-based nanoparticles and their applications in the delivery of bioactive compounds. Int. J. Food Prop..

[76] Zhang R., Han Y., Xie W., Liu F., Chen S. (2022). Advances in Protein-Based Nanocarriers of Bioactive Compounds: From Microscopic Molecular Principles to Macroscopical Structural and Functional Attributes. J. Agric. Food Chem..

[77] Nussbaum N., Bergfreund J., Vialetto J., Isa L., Fischer P. (2022). Microgels as globular protein model systems. Colloids Surf. B Biointerfaces.

[78] Kew B., Holmes M., Liamas E., Ettelaie R., Connell S.D., Dini D., Sarkar A. (2023). Transforming sustainable plant proteins into high performance lubricating microgels. Nat. Commun..

[79] Matalanis A., Jones O.G., McClements D.J. (2011). Structured biopolymer-based delivery systems for encapsulation, protection, and release of lipophilic compounds. Food Hydrocoll..

[80] Letocha A., Miastkowska M., Sikora E. (2022). Preparation and Characteristics of Alginate Microparticles for Food, Pharmaceutical and Cosmetic Applications. Polymers.

[81] McClements D.J. (2018). Encapsulation, protection, and delivery of bioactive proteins and peptides using nanoparticle and microparticle systems: A review. Adv. Colloid. Interface Sci..

[82] Wang Y., Zhao Y., He J., Sun C., Lu W., Zhang Y., Fang Y. (2023). Doubling growth of egg-box structure during Calcium-mediated molecular assembly of alginate. J. Colloid. Interface Sci..

[83] Sriamornsak P. (2003). Chemistry of pectin and its pharmaceutical uses: A review. Silpakorn Univ. Int. J..

[84] Necas J., Bartosikova L. (2013). Carrageenan: A review. Vet. Med..

[85] Jiang W., Zhai S., Zhu L., Bai Y., Li J., Li J. (2024). Protein/polysaccharide based oral delivery system for precise targeting of polyphenols and carotenoids. Food Biosci..

[86] Kumari A., Singh B. (2025). Emerging trends in designing polysaccharide based mucoadhesive network hydrogels as versatile platforms for innovative delivery of therapeutic agents: A review. Int. J. Biol. Macromol..

[87] Yousefi G., Farjadian S., Bour M.S.B., Hamedi A. (2025). Mucoadhesion, Rheology, Swelling Behavior, and Immunomodulatory Properties of Polysaccharides of Gum Exudates Obtained from *Astracantha echidnaeformis* (Sirj.) Podlech as a Formulary Excipient Candidate. Starch—Stärke.

[88] Sun L., Zhao J., Chen W., Wang G. (2025). Electrostatically sprayed alginate-carboxymethyl chitosan microgel for colon-targeted melatonin delivery ameliorates ulcerative colitis. Food Biosci..

[89] Li X., Tiang M.F., Cui X., Li Y., Wang Z., Zhao L., Takriff M.S., Sajab M.S., Abdul P.M., Ding G. (2024). Precisely controlled electrostatically sprayed sodium alginate/carboxymethyl chitosan hydrogel microbeads as super-adsorbent for adsorption of cationic dye. Int. J. Biol. Macromol..

[90] Liu M., Chen B., Ren F., Mitra P., Han Z., Zhu X., Huang M., Liu H. (2026). Protein-Polysaccharide Complexes: From Structure and Function to Food Applications—A Review. Food Rev. Int..

[91] Philip A.K., Samuel B.A., Saleh Y.S., Bhatia S., Mohammad B.I., Al-Aubaidy H.A. (2025). pH-responsive agarose hydrogel for enhanced gastrointestinal ibuprofen delivery: A novel pressure-sensitive adhesive system. Next Mater..

[92] Bhardwaj I., Ansari A.H., Rai S.P., Singh D., Singh S. (2025). Bioactive-Based Nanocarriers for Ocular Application. Bioact.-Based Nanother..

[93] Campagnoli L.I., Varesi A., Barbieri A., Marchesi N., Pascale A. (2023). Targeting the Gut–Eye Axis: An Emerging Strategy to Face Ocular Diseases. Int. J. Mol. Sci..

[94] Wang N., Luo L., Yang X. (2026). The gut-eye axis in age-related macular degeneration: From microbial dysbiosis to targeted intervention strategies. Exp. Biol. Med..

[95] Ahmadzadeh S., Ubeyitogullari A. (2023). Enhancing the stability of lutein by loading into dual-layered starch-ethyl cellulose gels using 3D food printing. Addit. Manuf..

[96] Kirtil E., Yildiz E. (2025). Recent advances and future frontiers in Pickering emulsions for food applications: Bridging science, industry, and nutrition. Food Res. Int..

[97] Cassani L., Gomez-Zavaglia A. (2024). Pickering emulsions in food and nutraceutical technology: From delivering hydrophobic compounds to cutting-edge food applications. Explor. Foods Foodomics.

[98] Yang J., Zhu B., Lu K., Dou J., Ning Y., Wang H., Li Y., Qi B., Jiang L. (2023). Construction and characterization of Pickering emulsions stabilized by soy protein hydrolysate microgel particles and quercetin-loaded performance in vitro digestion. Food Res. Int..

[99] Horvát G., Budai-Szűcs M., Berkó S., Szabó-Révész P., Soós J., Facskó A., Maroda M., Mori M., Sandri G., Bonferoni M.C. (2015). Comparative study of nanosized cross-linked sodium-, linear sodium- and zinc-hyaluronate as potential ocular mucoadhesive drug delivery systems. Int. J. Pharm..

[100] Zhang P., Nie Y., Wang X., Zhang X., Liu L. (2026). Next-generation smart ophthalmic biomaterials: From passive response to active interaction and closed-loop control. Bioact. Mater..

[101] Wang H., Chen Y., Zhao A., Shen Z., Zhang Y. (2026). The role of probiotics in modulation of the gut-brain axis: A prospective therapy for depression and mood disorders. Front. Pharmacol..

[102] Martelli A., Mohamed Y.O.A., Gallego-Ferrer G., Gentile P., Girón-Hernández J. (2025). Revolutionizing gut health: Advances in encapsulation strategies for probiotics and bioactive molecules. Biotechnol. Adv..

[103] McClements D.J. (2017). Recent progress in hydrogel delivery systems for improving nutraceutical bioavailability. Food Hydrocoll..

[104] Chen L., Remondetto G.E., Subirade M. (2006). Food protein-based materials as nutraceutical delivery systems. Trends Food Sci. Technol..

[105] Karim A., Rehman A., Feng J., Noreen A., Assadpour E., Kharazmi M.S., Lianfu Z., Jafari S.M. (2022). Alginate-based nanocarriers for the delivery and controlled-release of bioactive compounds. Adv. Colloid. Interface Sci..

[106] Shahidi F., Athiyappan K.D. (2025). Polyphenol-polysaccharide interactions: Molecular mechanisms and potential applications in food systems—A comprehensive review. Food Prod. Process. Nutr..

[107] Brahmi M., Essifi K., Tahani A., Gharsallaoui A. (2025). Impact of pH on sodium caseinate binding and structural changes on montmorillonite surface. Int. J. Biol. Macromol..

[108] Liu K., Chen Y.Y., Pan L.H., Li Q.M., Luo J.P., Zha X.Q. (2022). Co-encapsulation systems for delivery of bioactive ingredients. Food Res. Int..

[109] Wen C., Tang J., Cao L., Fan M., Lin X., Liu G., Liang L., Liu X., Zhang J., Li Y. (2025). Strategic Approaches for Co-Encapsulation of Bioactive Compounds: Technological Advances and Mechanistic Insight. Foods.

[110] Xu X., Tang Q., Gao Y., Chen S., Yu Y., Qian H., McClements D.J., Cao C., Yuan B. (2025). Recent developments in the fabrication of food microparticles and nanoparticles using microfluidic systems. Crit. Rev. Food Sci. Nutr..

[111] Xiang G., Yin B., Shiroud Heidari B., Youssef G., Gosecka M., Gosecki M., Torres F.G., Wong S.H.D., Dodda J.M. (2025). Programmable Hydrogels: Frontiers in Dynamic Closed-Loop Systems, Biomimetic Synergy, and Clinical Translation. Adv. Sci..

[112] Segneanu A.-E., Bejenaru L.E., Bejenaru C., Blendea A., Mogoşanu G.D., Biţă A., Boia E.R. (2025). Advancements in Hydrogels: A Comprehensive Review of Natural and Synthetic Innovations for Biomedical Applications. Polymers.

[113] Menon M., Mohammadi S., Davila-Velderrain J., Goods B.A., Cadwell T.D., Xing Y., Stemmer-Rachamimov A., Shalek A.K., Love J.C., Kellis M. (2019). Single-cell transcriptomic atlas of the human retina identifies cell types associated with age-related macular degeneration. Nat. Commun..

[114] Wei Z., Wang S., Hirvonen J., Santos H.A., Li W. (2022). Microfluidics Fabrication of Micrometer-Sized Hydrogels with Precisely Controlled Geometries for Biomedical Applications. Adv. Healthc. Mater..

[115] Wassén S., Rondeau E., Sott K., Lorén N., Fischer P., Hermansson A.-M. (2012). Microfluidic production of monodisperse biopolymer particles with reproducible morphology by kinetic control. Food Hydrocoll..

[116] Herrero E.P., Valle E.M.M.D., Galán M.A. (2006). Development of a new technology for the production of microcapsules based in atomization processes. Chem. Eng. J..

[117] Zhu Y., Peng Y., Wen J., Quek S.Y. (2021). A Comparison of Microfluidic-Jet Spray Drying, Two-Fluid Nozzle Spray Drying, and Freeze-Drying for Co-Encapsulating beta-Carotene, Lutein, Zeaxanthin, and Fish Oil. Foods.

[118] Xu Y., Zhu H., Denduluri A., Ou Y., Erkamp N.A., Qi R., Shen Y., Knowles T.P.J. (2022). Recent Advances in Microgels: From Biomolecules to Functionality. Small.

[119] Alvim I.D., Stein M.A., Koury I.P., Dantas F.B.H., Cruz C.L.d.C.V. (2016). Comparison between the spray drying and spray chilling microparticles contain ascorbic acid in a baked product application. LWT.

[120] Lavoisier A., Vilgis T.A., Aguilera J.M. (2019). Effect of cysteine addition and heat treatment on the properties and microstructure of a calcium-induced whey protein cold-set gel. Curr. Res. Food Sci..

[121] Egan T., O’Riordan D., O’Sullivan M., Jacquier J.-C. (2014). Cold-set whey protein microgels as pH modulated immobilisation matrices for charged bioactives. Food Chem..

[122] Fu L., Ding F., Huang X., Zou X. (2025). Recent advances in polysaccharide-protein complex coacervates for food applications. Colloids Surf. B Biointerfaces.

[123] Papagiannopoulos A., Sotiropoulos K. (2022). Current Advances of Polysaccharide-Based Nanogels and Microgels in Food and Biomedical Sciences. Polymers.

[124] Glomm W.R., Molesworth P.P., Sandru E.M., Truong L.T., Brunsvik A., Johnsen H. (2021). Microencapsulation of Peppermint Oil by Complex Coacervation and Subsequent Spray Drying Using Bovine Serum Albumin/Gum Acacia and an Oxidized Starch Crosslinker. Appl. Sci..

[125] Udoetok I.A., Mohamed M.H., Wilson L.D. (2024). Stabilization of Oil-in-Water Pickering Emulsions by Surface-Functionalized Cellulose Hydrogel. Gels.

[126] Roosen J., Pype J., Binnemans K., Mullens S. (2015). Shaping of Alginate–Silica Hybrid Materials into Microspheres through Vibrating-Nozzle Technology and Their Use for the Recovery of Neodymium from Aqueous Solutions. Ind. Eng. Chem. Res..

[127] Wei Y., Liu Z., Guo A., Mackie A., Zhang L., Liao W., Mao L., Yuan F., Gao Y. (2021). Zein Colloidal Particles and Cellulose Nanocrystals Synergistic Stabilization of Pickering Emulsions for Delivery of β-Carotene. J. Agric. Food Chem..

[128] Deng Z., Jung J., Simonsen J., Zhao Y. (2018). Cellulose nanocrystals Pickering emulsion incorporated chitosan coatings for improving storability of postharvest Bartlett pears (*Pyrus communis*) during long-term cold storage. Food Hydrocoll..

[129] Jie Y., Chen F. (2022). Progress in the Application of Food-Grade Emulsions. Foods.

[130] Guo J., Jiang J., Gu X., Li X., Liu T. (2021). Encapsulation of β-carotene in calcium alginate hydrogels templated by oil-in-water-in-oil (O/W/O) double emulsions. Colloids Surf. A Physicochem. Eng. Asp..

[131] Levy R., Okun Z., Shpigelman A. (2022). Utilizing high-pressure homogenization for the production of fermented plant-protein yogurt alternatives with low and high oil content using potato protein isolate as a model. Innov. Food Sci. Emerg. Technol..

[132] Wang C., Wang J., Zhu D., Hu S., Kang Z., Ma H. (2020). Effect of dynamic ultra-high pressure homogenization on the structure and functional properties of whey protein. J. Food Sci. Technol..

[133] Gharsallaoui A., Roudaut G., Chambin O., Voilley A., Saurel R. (2007). Applications of spray-drying in microencapsulation of food ingredients: An overview. Food Res. Int..

[134] Desai K.G.H., Jin Park H. (2005). Recent Developments in Microencapsulation of Food Ingredients. Dry. Technol..

[135] Masters K. (1991). Spray Drying Handbook.

[136] Quek S.Y., Chok N.K., Swedlund P. (2007). The physicochemical properties of spray-dried watermelon powders. Chem. Eng. Process. Process Intensif..

[137] Zambrano-Zaragoza M.L., Mendoza-Muñoz N., Urbán-Morlán Z., Quintanar-Guerrero D., Leyva-Gómez G., Gopi S., Balakrishnan P., Bračič M. (2022). Food-grade Biopolymers as Platforms for Nutrient Delivery. Biopolymers in Nutraceuticals and Functional Foods.

[138] Oh J.K., Lee D.I., Park J.M. (2009). Biopolymer-based microgels/nanogels for drug delivery applications. Prog. Polym. Sci..

[139] Xu Y., Yan X., Zheng H., Li J., Wu X., Xu J., Zhen Z., Du C. (2024). The application of encapsulation technology in the food Industry: Classifications, recent Advances, and perspectives. Food Chem. X.

[140] Ji R., Wu J., Zhang J., Wang T., Zhang X., Shao L., Chen D., Wang J. (2019). Extending Viability of Bifidobacterium longum in Chitosan-Coated Alginate Microcapsules Using Emulsification and Internal Gelation Encapsulation Technology. Front. Microbiol..

[141] Yao Y., Lin J.J., Chee X.Y.J., Liu M.H., Khan S.A., Kim J.E. (2021). Encapsulation of Lutein via Microfluidic Technology: Evaluation of Stability and In Vitro Bioaccessibility. Foods.

[142] Agriopoulou S., Smaoui S., Chaari M., Varzakas T., Can Karaca A., Jafari S.M. (2024). Encapsulation of Probiotics within Double/Multiple Layer Beads/Carriers: A Concise Review. Molecules.

[143] Minelgaitė V., Jeznienė S., Šipailienė A. (2025). Advances in food-grade hydrogel encapsulation of probiotics with next-generation prebiotics for targeted synbiotic delivery. Food Hydrocoll. Health.

[144] Álvarez-Henao M.V., Saavedra N., Medina S., Jiménez Cartagena C., Alzate L.M., Londoño-Londoño J. (2018). Microencapsulation of lutein by spray-drying: Characterization and stability analyses to promote its use as a functional ingredient. Food Chem..

[145] Kaushik P., Dowling K., Barrow C.J., Adhikari B. (2015). Microencapsulation of omega-3 fatty acids: A review of microencapsulation and characterization methods. J. Funct. Foods.

[146] Zhang Z., Zhang R., Tong Q., Decker E.A., McClements D.J. (2015). Food-grade filled hydrogels for oral delivery of lipophilic active ingredients: Temperature-triggered release microgels. Food Res. Int..

[147] Liu L., McClements D.J., Liu X., Liu F. (2024). Overcoming Biopotency Barriers: Advanced Oral Delivery Strategies for Enhancing the Efficacy of Bioactive Food Ingredients. Adv. Sci..

[148] Wang X., Gao S., Yun S., Zhang M., Peng L., Li Y., Zhou Y. (2022). Microencapsulating Alginate-Based Polymers for Probiotics Delivery Systems and Their Application. Pharmaceuticals.

[149] Sun R., Lv Z., Wang Y., Gu Y., Sun Y., Zeng X., Gao Z., Zhao X., Yuan Y., Yue T. (2024). Preparation and characterization of pectin-alginate-based microbeads reinforced by nano montmorillonite filler for probiotics encapsulation: Improving viability and colonic colonization. Int. J. Biol. Macromol..

[150] Li Q., Duan M., Hou D., Chen X., Shi J., Zhou W. (2021). Fabrication and characterization of Ca(II)-alginate-based beads combined with different polysaccharides as vehicles for delivery, release and storage of tea polyphenols. Food Hydrocoll..

[151] Rezaei A., Rafieian F., Akbari-Alavijeh S., Kharazmi M.S., Jafari S.M. (2022). Release of bioactive compounds from delivery systems by stimuli-responsive approaches; triggering factors, mechanisms, and applications. Adv. Colloid. Interface Sci..

[152] Hua Z., Zhang X., Su W., Zaky A.A., El-Aty A.M.A., Xu E., Liu D., Tan M. (2026). Advancements in precision nutrition: Targeted strategies for personalized health. Innov. Life.

[153] Yang K., Han H.S., An S.H., Park K.H., Nam K., Hwang S., Lee Y., Cho S.Y., Kim T., Choe D. (2024). Mucoadhesive chitosan microcapsules for controlled gastrointestinal delivery and oral bioavailability enhancement of low molecular weight peptides. J. Control. Release.

[154] Saberi Riseh R., Skorik Y.A., Thakur V.K., Moradi Pour M., Tamanadar E., Noghabi S.S. (2021). Encapsulation of Plant Biocontrol Bacteria with Alginate as a Main Polymer Material. Int. J. Mol. Sci..

[155] Ghiasi F., Hashemi H., Esteghlal S., Hosseini S.M. (2024). An Updated Comprehensive Overview of Different Food Applications of W1/O/W2 and O1/W/O2 Double Emulsions. Foods.

[156] Liu L., Yao W., Rao Y., Lu X., Gao J. (2017). pH-Responsive carriers for oral drug delivery: Challenges and opportunities of current platforms. Drug Deliv..

[157] Boon C.S., McClements D.J., Weiss J., Decker E.A. (2010). Factors influencing the chemical stability of carotenoids in foods. Crit. Rev. Food Sci. Nutr..

[158] McClements D.J., Decker E.A. (2000). Lipid oxidation in oil-in-water emulsions: Impact of molecular environment on chemical reactions in heterogeneous food systems. J. Food Sci..

[159] Shewan H.M., Stokes J.R. (2013). Review of techniques to manufacture micro-hydrogel particles for the food industry and their applications. J. Food Eng..

[160] Karimi I., Mohammed L.J., Amshawee A.M., Makki N.F., Nazari K., Schioth H.B. (2025). Mannans as Multifunctional Biopolymers: Structure, Properties, and Applications in Health and Industry. Polymers.

[161] Shehzad Q., Rehman A., Jafari S.M., Zuo M., Khan M.A., Ali A., Khan S., Karim A., Usman M., Hussain A. (2021). Improving the oxidative stability of fish oil nanoemulsions by co-encapsulation with curcumin and resveratrol. Colloids Surf. B Biointerfaces.

[162] Pouton C.W., Porter C.J. (2008). Formulation of lipid-based delivery systems for oral administration: Materials, methods and strategies. Adv. Drug Deliv. Rev..

[163] Group A.R., Chew E.Y., Clemons T., SanGiovanni J.P., Danis R., Domalpally A., McBee W., Sperduto R., Ferris F.L. (2012). The Age-Related Eye Disease Study 2 (AREDS2): Study design and baseline characteristics (AREDS2 report number 1). Ophthalmology.

[164] Gautam M., Santhiya D. (2019). Pectin/PEG food grade hydrogel blend for the targeted oral co-delivery of nutrients. Colloids Surf. A Physicochem. Eng. Asp..

[165] McClements D.J. (2010). Design of Nano-Laminated Coatings to Control Bioavailability of Lipophilic Food Components. J. Food Sci..

[166] Frestedt J.L., Holban A.M., Grumezescu A.M. (2018). Chapter 16—Foods, Food Additives, and Generally Regarded as Safe (GRAS) Food Assessments. Food Control and Biosecurity.

[167] de Boer A., Bast A. (2018). Demanding safe foods—Safety testing under the novel food regulation (2015/2283). Trends Food Sci. Technol..

[168] Turck D., Bresson J.-L., Burlingame B., Dean T., Fairweather-Tait S., Heinonen M., Hirsch-Ernst K.I., Mangelsdorf I., McArdle H., EFSA Panel on Dietetic Products Nutrition and Allergies (2016). Guidance on the preparation and presentation of an application for authorisation of a novel food in the context of Regulation (EU) 2015/2283. EFSA J..

[169] Faustino M., Veiga M., Sousa P., Costa E.M., Silva S., Pintado M. (2019). Agro-Food Byproducts as a New Source of Natural Food Additives. Molecules.

[170] Johnson E.J. (2014). Role of lutein and zeaxanthin in visual and cognitive function throughout the lifespan. Nutr. Rev..

[171] Trieschmann M., van Kuijk F.J.G.M., Alexander R., Hermans P., Luthert P., Bird A.C., Pauleikhoff D. (2008). Macular pigment in the human retina: Histological evaluation of localization and distribution. Eye.

[172] Beatty S., Boulton M., Henson D., Koh H.H., Murray I.J. (1999). Macular pigment and age related macular degeneration. Br. J. Ophthalmol..

